# Deep sequencing of transcriptome profiling of *GSTM2* knock-down in swine testis cells

**DOI:** 10.1038/srep38254

**Published:** 2016-12-01

**Authors:** Yuqi Lv, Yi Jin, Yongqiang Zhou, Jianjun Jin, Zhenfa Ma, Zhuqing Ren

**Affiliations:** 1 Key Laboratory of Swine Genetics and Breeding of Ministry of Agriculture & Key Laboratory of Agriculture Animal Genetics, Breeding and Reproduction of Ministry of Education, College of Animal Science, Huazhong Agricultural University, Wuhan, 430070 Hubei, P.R. China; 2 The Cooperative Innovation Center for Sustainable Pig Production, Huazhong Agricultural University, Wuhan, 430070 Hubei, P.R. China

## Abstract

Glutathione-S-transferases mu 2 (*GSTM2*), a kind of important Phase II antioxidant enzyme of eukaryotes, is degraded by nonsense mediated mRNA decay due to a C27T substitution in the fifth exon of pigs. As a reproductive performance-related gene, *GSTM2* is involved in embryo implantation, whereas, functional deficiency of *GSTM2* induces pre- or post-natal death in piglets potentially. To have some insight into the role of *GSTM2* in embryo development, high throughput RNA sequencing is performed using the swine testis cells (ST) with the deletion of *GSTM2*. Some embryo development-related genes are observed from a total of 242 differentially expressed genes, including *STAT1, SRC, IL-8, DUSP* family, *CCL* family and integrin family. *GSTM2* affects expression of *SRC, OPN,* and *SLC*s. GSTM2 suppresses phosphorylation of STAT1 by binding to STAT1. In addition, as an important transcription factor, *STAT1* regulates expression of uterus receptive-related genes including *CCLs, IRF9, IFITs, MXs, and OAS*. The present study provides evidence to molecular mechanism of *GSTM2* modulating embryo development.

Glutathione S-transferases (GSTs), as members of a supergene family of multifunctional enzymes, play an important role in the defense to a wide array of toxic and carcinogenic substances^1^,^2^ by catalyzing the conjugation of glutathione (GSH) with a broad range of electrophilic compounds. Up to now, eight distinct classes of GSTs have been identified^1^, whereas, different class of GST enzymes have different functions. Mu class (GSTMs) are mainly involved in eliminations of free radicals, peroxides, electrophilic reagents, heavy metals, also, they mediates and regulates cells protection and growth. Among the members of GSTMs, *GSTM2* is a potential candidate involved in reproductive regulation due to high expression level in spermaduct, epididymis, testis, ovary, and oviduct, which was mentioned by a study for mammalian reproduction^2^. It is reported that ova resists the endogenous and exogenous toxic substances by *GSTM2* in ovary^3^, which characterizes *GSTM2* as a protector for germ cells. *GSTM2* participates in the generation of prostaglandin E2 (PGE2)^4^ that is essential for testis maturation and embryo implantation^5^,^6^,^7^,^8^. *GSTM2* is up-regulated forcefully in luminal epithelium of uterine at the day 3 and day 4 after pregnancy^9^, and additionally, progesterone is probably involved in up-regulation of *GSTM2*, which shows the necessity of *GSTM2* in the preparation of uterine in blastocyst implantation process^9^. Interestingly, the high expression of *GSTM2* in progression of embryonic reactivation^10^ suggests the potential effect on embryo development.

In a previous study of our lab, it has been identified that a premature translation termination codon (PTC) caused by a nonsense mutation (CGA→TGA) resulting from a C27T substitution in the fifth exon of *GSTM2*. Nonsense-mediated mRNA decay (NMD) could degrade the mutated porcine *GSTM2* mRNA^11^ because of the specifical identification and degradation of aberrant transcripts harboring a premature termination codon (PTC)^12^,^13^. Interestingly, the homozygous genotype TT was not found in 164 individuals from Large White, Landrace, Meishan and Qingping pigs^11^. The embryo with a *GSTM2* TT genotype would be most likely to die or abort. To give insight into the role of *GSTM2* in embryo development, RNA-seq was performed from ST cells treated with siRNAs targeting *GSTM2*.

## Results

### Small interfering RNA treatment repressed *GSTM2* in swine testis cells

Three pairs of siRNAs named si1, si2, and si3, were designed to suppress expression of GSTM2 in ST cells. The mRNA and protein level of GSTM2 was decreased significantly (*P* < *0.01*) at 24 h after transfection (Supplementary Fig. S1a), and furthermore, si2 worked best for the suppression (Supplementary Fig. S1b).

### Sequence quality and saturation analysis

RNA integrity was assessed by BioAnalyzer in the present study. The RIN value of all samples were over 7. The raw data which contained adaptor sequences were transformed into clean tags. Preparation and experiments of sample RNA were thought to be convincing if the tags contains N were less than 10% of total raw data, and copy number less than 2 tags was no more than 20%. The data showed that all the samples conformed to sequencing requirement and the experiments were successful (Supplementary Fig. S2a). In addition, the repeatability of RNA-seq was tested (Supplementary Fig. S2b). R values both from Spearman and Pearson analyses over 8 showed a high repeatability between two samples. The saturation analysis could be performed to check whether the number of detected genes keep increasing when sequencing amount (total tag number) increases. The data showed that when sequencing amount reaches 2 M or higher, the number of detected genes almost ceases to increase (Supplementary Fig. S2c). Thereby, the result indicated that the high-throughput Illumina sequencing data were exhaustive.

### Data analysis of RNA-seq

In the present study, six cDNA libraries (TR1, TR2, TR3, CK1, CK2 and CK3) were established by reverse transcription of total RNA from three wells of treated ST cells (with siRNA2) and three wells of un-treated ST cells respectively. The standard analyses for quality control^14^ was conducted to ensure the quality of RNA met the requirement for sequencing (Supplementary Fig. S3). A total of 11,927,452 (98.78%), 11,865,576 (99.01%), 11,870,090 (98.74%) clean reads were obtained from three treatment groups respectively. 11,790,005 (98.79%), 12,129,113 (98.75%), and 12,192,917 (98.85%) clean reads were obtained from three control groups respectively (Supplementary Table S2a). A total of 22,966,999 reads of treatment groups and 24,559,030 reads of control groups were mapped to reference gene. A total of 26,394,293 reads of treatment groups and 27,449,663 reads of control groups were mapped to reference genome respectively (Supplementary Table S2b). The raw data was obtained from transformed sequence data by Base Calling, and then the raw reads that contained adaptor sequence data and N > 10% were removed. Furthermore sequence data were filtered for low-quality reads at high level of stringency. After these steps, approximately 99% reads of the raw data were clean reads, whereas only 1% of the data came from adaptor sequences attached to empty vectors. The clean reads were mapped to the reference gene and the reference genome of pig (Supplementary Table S2b and Supplementary Table S3). The evaluation of gene expression levels were displayed in reads per kilo base of gene per million reads (RPKM)^15^. We used a standard to classify genes into three expression levels, including low expression (0 < RPKM < 5), moderate expression (10 < RPKM < 50), and high expression (RPKM > 500).

### Differentially expressed genes (DEGs) between treatment groups and control groups

**Figure 1:**
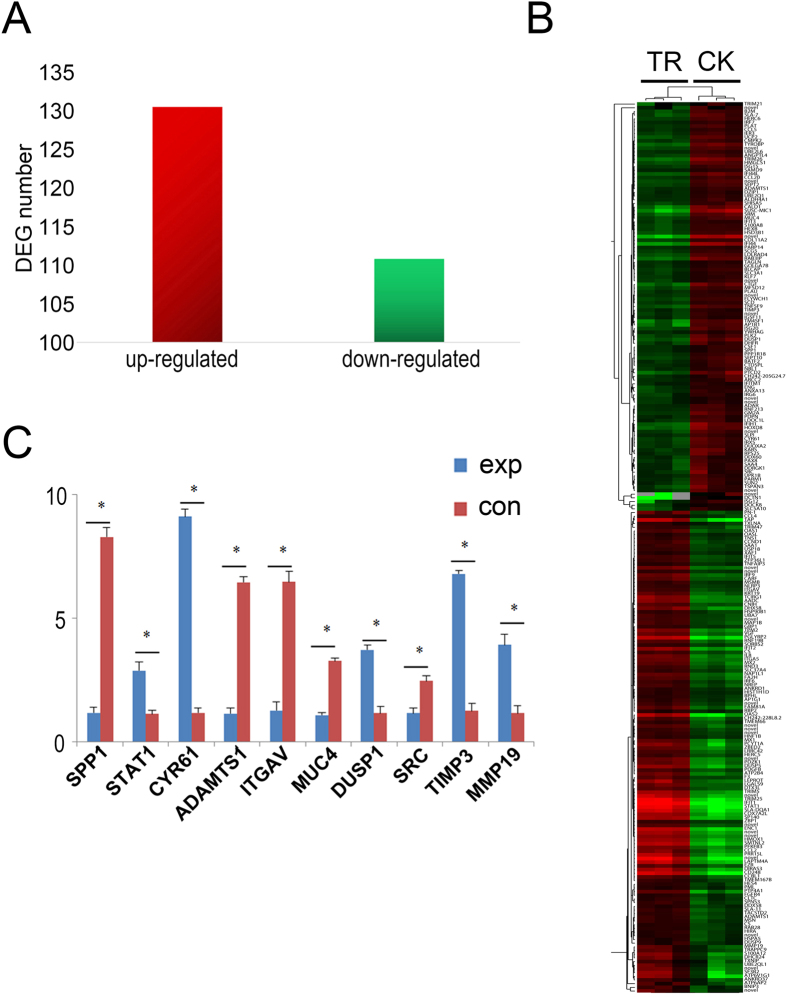
Analysis of differentially expressed genes. (**A**) DEGs (FDR ≤ 0.001 and |log2 Ratio| ≥ 1) identified between TR and CK. (**B**) The heatmap of the subset DEGs in different samples (TR1, TR2, TR3, CK1, CK2, CK3). (**C**) Several DEGs related to embryo development from RNA-seq data were screened for validation by qRT-PCR.

Total of 242 different transcripts within 131 up-regulated DEGs and 111 down-regulated DEGs were identified through the EdgeR analysis (|log2Raito| ≥ 1, FDR ≤ 0.001) in treatment groups compared to control group (Fig. 1 and Supplementary Table S4).

### Verification of DEGs with quantitative real-Time PCR (qRT-PCR)

Several DEGs related to embryo development from RNA-seq data were screened for validation by qRT-PCR (Fig. 1C). The results showed that the expression of *SPP1, ADAMTS1, ITGAV, MUC4,* and *SRC* was down-regulated, whereas *STAT1, CYR61, DUSP1, TIMP3* and *MMP19* was up-regulated, which were consistent with the expression profile of RNA-seq.

### Gene ontology and KEGG pathway enrichment analysis for DEGs

**Figure 2:**
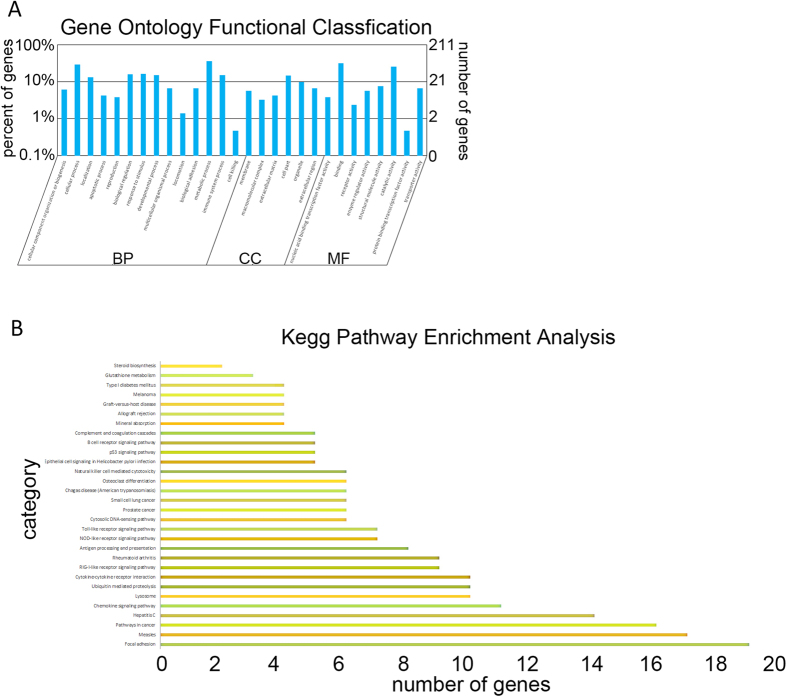
Histogram of Gene Ontology (GO) enrichment and pathway analysis of DEGs. (**A**) All the GO terms are summarized in three main categories which are biological process, cellular component and molecular function. (**B**) The differentially expressed genes were clustered and enriched in several pathways including focal adhesion, measies, pathways in cancer, hepatitis C, chemokine signaling pathway, and etc.

GO classification of the DEGs revealed that 110 genes are involved in metabolic processes, 37 in stimuli, 38 in immune system processes, and 13 in biological adhesion, respectively (Fig. 2A). These 242 DEGs mainly participated in over 30 pathways according to PANTER, including inflammation mediated by chemokine and cytokine signaling pathways, integrin signaling pathways, Parkinson’s disease, angiogenesis, gonadotropin releasing hormone receptor pathway, interleukin signaling pathway, EGF receptor signaling pathway, FGF signaling pathway etc. (Fig. 2B). Detail information of GO and pathway analysis was showed in Supplementary Table S5 and Supplementary Table S6, respectively.

### Genes involved in the Maternal-Placental Interface and embryonic development

The knockdown of *GSTM2* in ST cells alerts the expression of some genes involved in maternal-placental interface and embryonic development. Three SLC family genes (*SLC5A10, SLC3A1*, and *SLC37A4*) were down-regulated in GSTM2 knock-down ST cells. Another down-regulated DEGs is FGFR4, which is a member of the fibroblast growth factor receptor (FGFR) family (Supplementary Fig. S4A). Furthermore, Six genes related to the process of cell adhesion were down-regulated in the *GSTM2* knock-down ST cells, including tetraspanin 3 (*TSPAN3*), a disintegrin and metalloproteinase with thrombospondin motifs 1 (*ADAMTS1*), integrin, alpha V (*ITGAV*), GTP-binding RAS-like 3 (*DIRAS3*), immunoglobulin superfamily 11 (*IGSF11*) and secreted phosphoprotein 1 (*SPP1*, also known as osteopontin (*OPN*)) (Supplementary Fig. S4B). More than 30 IFN-stimulated genes were up-regulated, including ISG family, IFIT, IRF, MX *etc.* (Supplementary Fig. S4C). In addition, IFN-stimulated genes, some cytokines and chemokines in the endometrium related to implantation, including *CCL2, CCL4, CCL20, CCL5*, and *CXCL8*^16^,^17^,^18^,^19^, were also up-regulated. Another up-regulated gene, *CSF1*, is a specific hemopoietic growth factor regulating survival, proliferation and differentiation of mononuclear phagocytes.

### Suppression of STAT1 phosphorylation by overexpression of *GSTM2*

**Figure 3:**
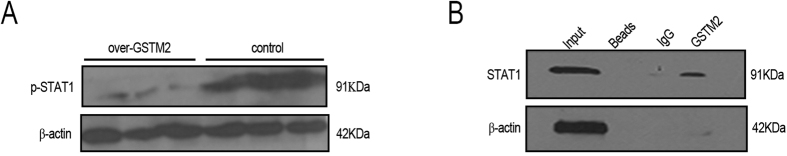
GSTM2 suppressed STAT1 phosphorylation by binding STAT1. (**A**) Detection of phosphorylation of STAT1 after the overexpression of *GSTM2*. (**B**) Co-immunoprecipitation experiment detected the combination between GSTM2 and STAT1. β-actin was used as internal control. *P < 0.05, **P < *0.01*.

In order to provide more insight into relationship between GSTM2 and STAT1, we overexpressed *GSTM2* in ST cells. Protein level of *STAT1* was decreased (Supplementary Fig. S5A,C) when *GSTM2* was overexpressed. In addition, the mRNA expression level of downstream targets of STAT1 including *ITGAV, SRC*, and *OPN* were up-regulated (Supplementary Fig. S5B). Overexpression of *GSTM2* suppressed the phosphorylation of STAT1 (Fig. 3A). Furthermore, co-immunoprecipitation assay indicated that GSTM2 could bind to STAT1 (Fig. 3B).

### Overexpression and interference assay of *STAT1*

**Figure 4:**
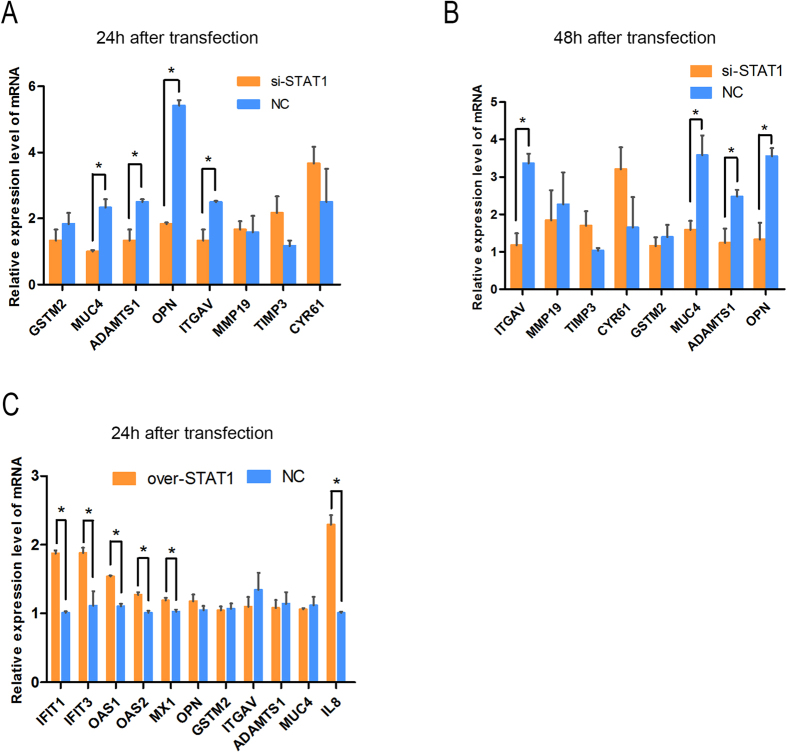
Knockdown and overexpression experiment of STAT1 in ST cells. (**A**) Knockdown *STAT1* in ST cells using siRNA and detect downstream genes at 24 h using q-PCR. (**B**) Knockdown *STAT1* in ST cells using siRNA and detect downstream genes at 48 h using q-PCR. (**C**) Overexpress STAT1 in ST cells and detect downstream genes at 24h using q-PCR. *P < 0.05.

*STAT1* was knocked down in ST cells by RNAi (Supplementary Figure S6 A–D). The mRNA expression of *MUC4, ADAMTS1, OPN*, and *ITGAV*, which belong to downstream targets of *STAT1*, were decreased (*P* < 0.05, Fig. 4A,B), but did not have a significant change (*P* > 0.05) when *STAT1* was overexpressed (Supplementary Figure S6 E,F). *IFIT1, IFIT3, MX1, OAS1, OAS2*, and immune-related genes were up-regulated (*P* < *0.05*, Fig. 4C) in the presence of overexpression of *STAT1*.

### Expression profile of *GSTM2* and *STAT1* in porcine endometrium

**Figure 5:**
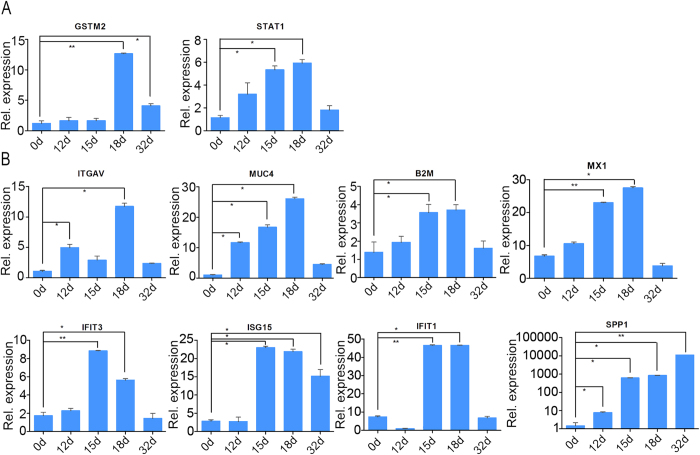
Temporal expression profiles of interesting genes. Expression level of *GSTM2, STAT1, SPP1, ITGAV, MUC4, IFIT3, ISG15, B2M, IFIT1*, and *MX1* in porcine endometrium. Day 0, 12, 15, 18, and 32 were selected for the detection. *P < 0.05, **P < 0.01.

The expression level of *GSTM2, STAT1* and some other DEGs was detected in porcine endometrium with different developmental stages, including day 0, day 12, day 15, day 18 and day 32 of gestation stages. The expression level of *GSTM2* was increased at day 18 and day 32 compared to day 0 and day 12, whereas the *STAT1* was increased at day 12, day 15, and day 18 gradually compared to 0d. Both *GSTM2* and *STAT1* had a highest expression level at day 18, whereas, the expression sharply decreased at day 32 (Fig. 5A). The expression level of downstream targets of *STAT1* including immune-related genes *IFIT1, IFIT3, ISG15, B2M*, and adhesion process-related genes *SPP1, MUC4, ITGAV* and *MX1,* was also detected. Interestingly, all these genes expressed abundantly at day 15 and day 18 compared to day 0 and day 12 except for *ITGAV*, whereas, expression of *SPP1, MUC4* and *ITGAV* began to increase at day 12. Furthermore, the expression of *ISG15* and *SPP1* was still high at day 32, whereas the others were resumed to the expression level of day 0. The expression pattern of *MUC4, B2M* and *MX1* was similar to that of *STAT1* (Fig. 5B).

## Discussion

As one kind of important phase II antioxidant enzymes *in vivo,* GSTs were a wide array of toxic and carcinogenic substances. In this superfamily, mu class of GST could eliminate free radicals, peroxides, electrophilic reagents, heavy metals, and mediate cell protection and the regulation of cell growth. *GSTM2*, a member of GST mu class, was widely and highly expressed in various tissues including embryos, and testis^11^. In gestation stage of mice, *GSTM2* expressed at a low level in luminal epithelium at day 3, but expressed highly at day 4 during early pregnancy^9^. In a previous study from our lab, the NMD-induced degradation of *GSTM2* was most likely to cause embryonic death or abortion. Furthermore, this genotype that could be degraded by NMD was not found in 164 adults from Large White, Landrace, Meishan and Qingping pigs^11^. Here we knocked down *GSTM2* by small interference RNAs *in vitro* to simulate the degradation of *GSTM2 in vivo*. High-throughput sequencing with the *GSTM2*-knockdown ST cells would be helpful to deeply understand the function of GSTM2.

The transcriptome data obtained from the present study were useful for the elucidation of function of GSTM2. Usually more than 18M reads from RNA-seq analyses are required for each sample to attain a saturated state for novel gene discovery and expressional analysis^14^,^20^. The quality control of our data revealed that the RNA-seq data were well qualified (Supplementary Table S2).

Bioinformatics analyses of DEGs discovered DEGs between the *GSTM2*-knockdown group and control group. After filtering with the standard, 242 DEGs were obtained from there six samples (Fig. 1B). Some down-regulated genes, including *TSPAN3, ADAMTS1, ITGAV, DIRAS3, IGSF11* and *SPP1*, were involved in embryo implantation. Among them, TSPAN3 as a member of transmembrane 4 superfamily (TM4SF) involved in the adhesion, migration, perliferation, differentiation and signal transduction of cells^21^ has a high expression at the blastocyst of *Xenopus Laevis*, and furthermore played an important role in the communication between blastocyst and endometrium in mammals^22^,^23^,^24^ especially in cow^25^.

According to our data, three SLC family genes (*SLC5A10, SLC3A1*, and *SLC37A4*), one FGFR family gene (*FGFR4*), and six cell adhesion-related genes (*TSPAN3, ADAMTS1, ITGAV, DIRAS3, IGSF11*, and *OPN*) were down-regulated in GSTM2-knockdown ST cells. Whereas, a lot of IFN-stimulated genes including ISG family, *IFIT, IRF, MX*, and some cytokines/chemokines including *CCL2, CCL4, CCL20, CCL5,* and *CXCL8*^16^,^17^,^18^,^19^ were up-regulated. Among these genes, OPN was a powerful gene involved in embryo implantation. The expression of *OPN* was modulated by estrogen in pregnancy^26^. *OPN* is also one component of the extracellular matrix (ECM) serving as an integrin ligand. Additionally, it was expressed in a small number of stroma cells at day 9 of pregnancy, the uterine LE adjacent to conceptus tissue beginning at day 12 and throughout the LE surface by day 20^27^. Although *OPN* was clustered to estrogen-responsible gene, the concrete regulation of estrogen on expression of *OPN* was indirect through its interactions with *ERα*^28^. The SRC family members enhanced the transcriptional activity of a variety of nuclear receptors, including *ERα, ERβ* and *PR*^29^,^30^,^31^,^32^. The expression of *GSTM2, SRC* and *OPN* was down-regulated significantly at the interface. Therefore, we suspected that *GSTM2* regulated the expression of *SRC* that affected the activity of *ERα* and resulted in embryo implantation by suppressing *OPN* expression. *OPN* expressed both in endometrial epithelial cells and in trophoblast cells due to the interaction between *OPN* and trophoblast expression of integrin. This interaction achieved embryo adhesion and early communication to mother directly. *OPN* binds to the receptors including *CD44* and integrins on the cell surface, and then initiated a variety of kinase cascades, including focal adhesion kinase (FAK) and phosphatidylinositol 3-kinase (PI3K)/apoptosis signal-regulating kinase 1 (AKT) signaling^33^,^34^,^35^. The increase of adhesion complex assembly in OPN treated blastocysts was mediated through FAK- and PI3K-dependent signaling pathways^26^. *GSTM2* was a downstream gene of *PI3K*^36^. Thus, knockdown *GSTM2* may affect embryo adhesion by modulating interactions between *OPN* and integrins. *OPN* as a kind of ECM protein was degraded by *MMPs*. According to the data of RNA-seq, expression of *MMP19* was raised in the *GSTM2* knockdown group, which was likely to lower expression of *OPN*. However, the protease activity of *MMPs* was correlated with *TIMPs*, whereas, both of these proteins maintained the stability of the extracellular matrix. The expression of *TIMP3* gene was increased in the *GSTM2* knockdown group, whereas expression of *MMP19* was elevated as well. Only if the expression of *TIMPs/MMPs* was balanced, can embryo implantation be carried out. The knock-down of *GSTM2* may break the balance. Therefore we assumed that *GSTM2* repressed the activity of *ER via* the regulation of *SRC*, then the expression level of *OPN* changed.

IFN-stimulated genes and some cytokines/chemokines were identified from DEGs, but there was no evidence to prove that *GSTM2* could regulate these genes. STAT1 is involved in JAK-STAT pathway, which could be stimulated by some IFNs and cytokines, and regulates its downstream targets including some IFN-induced genes (IFNGs) and immune-related genes. Interestingly, previous study indicated that the interaction between GSTP1 and STAT3 regulated the phosphorylation of STAT3^37^. For this reason, we assumed that GSTM2 would regulate phosphorylation of STAT1. The result of co-IP experiment indicated that GSTM2 could bind to STAT1 (Fig. 3B), the phosphorylation level, furthermore, was reduced by the overexpression of *GSTM2* (Fig. 3A). It provided a better explanation for expression changes of downstream targets of STAT1.

We performed the overexpression and interference assay of *STAT1* to confirm whether these IFN-stimulated genes and some cytokines/chemokines were the downstream targets of *STAT1*. When *STAT1* was knocked down, the expression of *MUC4, ADAMTS1, OPN*, and *ITGAV* were decreased (*P* < 0.05, Fig. 4A,B), whereas, *IFIT1, IFIT3, MX1, OAS1* and *OAS2*, immune-related genes were up-regulated (*P* < 0.05, Fig. 4C) in the presence of overexpression of STAT1. Hence the activation of STAT1 would induce the up-regulation of IFNGs. IFNGs could enhance the uterus acceptance, however, the high expression of IFNGs would induce the blastocyst delay^38^. Maternal immune recognition of embryos occurs through two major pathways. One is that the immune system can detect the presence of alloantigens or receptor ligands on the conceptus. The other is that the immune system could be activated by chemokines and cytokines produced by the conceptus^39^. There were two classes of major histocompatibility complex (MHC) antigens, whereas, only MHC class I antigens were expressed on the surface of the conceptus. MHC class I molecules or transcripts could be detected throughout development to the blastocyst stage in cow^40^,^41^,^42^. However, at the period of placental attachment, little MHC antigens were expressed. Available evidence has indicated the down-regulation of MHC antigen class I in porcine trophoblast from days 14 to 25^43^. Some chemokines and cytokines, including interferons and CCL, activated the immune system, started the pregnancy recognition and protected against some bacteria during the peri-attachment period. Some of MHC genes were up-regulated with the knock-down of *GSTM2*, including MHC class I antigens *SLA7, SLA11* and SUSC-MIC1, and MHC class Π antigens SLA-DQA1. Porcine conceptus trophectoderm cells induced the expression of SLA class I and β2m genes through secretion of IFN-δ or IFN- γ in uterine stromal, but this expression was silenced in LE in order to prevent immune rejection at the uterine-placental interface^44^. SLA-DQ was expressed responding to IFN- γ from the conceptus. Additionally, it likely regulated immune responses at the maternal-fetal interface in order to support the maintenance of pregnancy in pigs^45^. Many IFN-stimulated genes were up-regulated, including *ISG* family, *IFIT, IRF, MX* etc. (Supplementary Fig. S4C), whereas the effects of expression of *Mx* were different in pregnancy recognition mechanism in the early pregnancy uterus of different domestic farm species^46^,^47^. Changes of related genes suggested that *GSTM2* was associated to immunological recognition of the conceptus. STAT1 participated in type I and type II interferon signaling pathways^48^,^49^. In the type I interferon activate signaling pathways, GSTM2 may be involved in IFNs signaling pathways by regulating STAT1.

**Figure 6:**
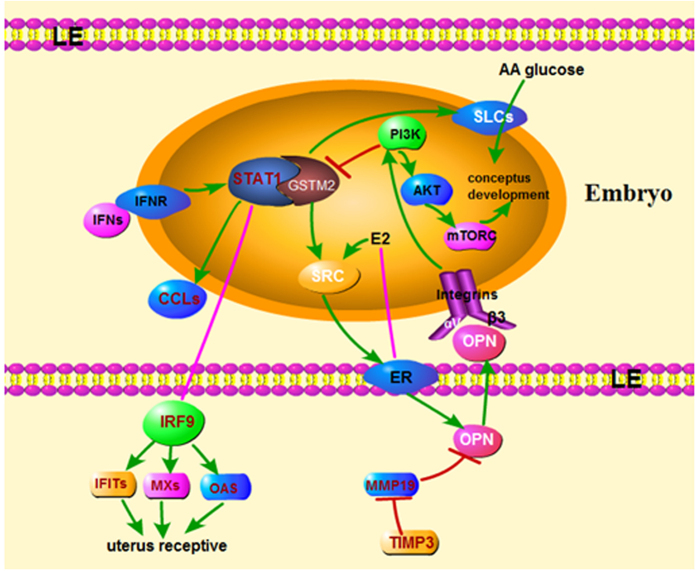
Potential network of GSTM2-STAT1 at the maternal-fetal interface in pigs. On the one hand, IFNs binds to IFNR and stimulates *STAT1*, whereas up-regulated STAT1 promotes the expression of *CCLs* and regulates uterus receptive through *IRF9* and the downstream targets *IFITs, MXs, OAS*. However, GSTM2 could bind to STAT1 and suppress the phosphorylation of STAT1. Hence, the high expression of these IFNGs which was regulated by *STAT1* would be balanced by *GSTM2*, so that the uterus receptive was promoted. On the other hand, *GSTM2* regulates OPN through SRC and ER, furthermore, OPN binds to integrin to regulate conceptus development through PI3K-AKT-mTORC signaling. *GSTM2* could also promote conceptus development by up-regulating expression of *SLCs* and increasing the AA and glucose into embryo.

We also detected the expression of related genes *in vivo*. The expression level of *GSTM2* was increased at day 18 and day 32 compared to day 0 and day 12, whereas the *STAT1* was increased at day 12, 15, and 18 gradually compared to day 0. The high level of *STAT1* at day 12 and day 15 did not induce the increase of expression of *GSTM2*. Both *GSTM2* and *STAT1* expressed highest at 18d, whereas, the expression sharply decreased at day 32 (Fig. 5A). Day 18 is essential time point for pig embryo implantation. Maybe more *GSTM2* was needed to suppress the effect of *STAT1*. The expression level of downstream targets of *STAT1* including immune-related genes *IFIT1, IFIT3, ISG15, B2M*, and adhesion process-related genes *SPP1, MUC4, MX1* expressed highly at day 15 and day 18 compared to day 0 and day 12, whereas, expression of *SPP1, MUC4* and *ITGAV* began to increase at day 12. Furthermore, the expression of *ISG15* and *SPP1* was still high at day 32, whereas the others were resumed to the expression level of day 0. The expression pattern of *MUC4, B2M* and *MX1* was similar to that of *STAT1* (Fig. 5B). A previous study about implantation showed that *GSTM2* was highly expressed during the early stage of implantation, and this condition just occurred in a very short period^9^. In addition, IFN was able to increase the capacity of the endometrium to adapt new conceptus, whereas overexpression of IFNG may induce delay of conceptus sometimes by 15–23 days^38^. In order to protect conceptus from the delay induced by IFNG, expression of *GSTM2* was up-regulated in the early stage of implantation. Porcine embryo implantation started at day 13 and finished at day 24 after pregnancy^50^. In general, we assumed that GSTM2-STAT1 promoted embryo implantation. Blastocyst attachment to the LE was only achieved by the transitional labilization and the remodeling of uterine epithelium polarity after the synchronous exchange of signals between the conceptus and endometrial cells^51^. Vascular permeability increases within 13 days of pig pregnancy^52^, whereas, by day 15 of pregnancy, IFNs up-regulated a large array of IFN induced genes in the underlying stroma and glandular epithelium, including *ISG15, IRF1, STAT1, SLAs* and *B2M*, which were likely to play roles in uterine remodeling to support placentation^53^. Another gene is F3, a tissue factor (TF) that existed in endometrial stromal cells and may play an important role during the period of embryo^54^. Matrix metalloproteinases (MMPs) are a family of zinc-dependent neutral endopeptidases that regulated tissue remodeling during embryonic development, angiogenesis and wound healing^55^,^56^,^57^. Among these, *MMP19* was expressed throughout the menstrual cycle, thereafter, affected cell proliferation and angiogenesis, which were crucial for endometrial receptivity^58^. Tissue inhibitor of matrix metalloproteinases (TIMPs) is an MMP inhibitor, and an abnormal balance between MMPs and TIMPs has been related to tumor invasion and metastasis in various human cancers, including endometrial cancers^59^,^60^. In general, we constructed a potential network of GSTM2-STAT1 at the maternal-fetal interface in pigs (Fig. 6).

## Methods

### Samples Collection and Animal Care Protocol

All animals were raised under the same conditions. We obtained endometrium of day 0, 12, 15, 18, and 32 clinically healthy gestation sow from the Fine Farm of HuaZhong Agricultural University. Tissue samples from endometrium were frozen in liquid nitrogen immediately after collection and stored at −80 °C prior to RNA extraction. The methods were performed in accordance with the approved guidelines from Huazhong Agricultural University, and scientific, ethical and legal principles of the Hubei Regulations for the Administration of Affairs Concerning Experimental Animals. All experimental protocols were approved by the Ethics Committee of Huazhong Agricultural University.

### Cell culture

Swine testis (ST) cells were purchased from CCTCC (China Center for Type Culture Collection, Wuhan, China) were cultured in Dulbecco’s modified Eagle’s medium (DMEM), supplemented with 10% (v/v) bovine calf serum (Gibico, USA) in a culture flask at 37 °C under a humidified atmosphere of 5% CO_2_. At 60–70% confluence, cells were trypsin-digested for further sub-culturing or seeded into 6-well plates (2 ml/well) at a concentration of 1–2 × 10^5^ cells per ml for siRNA transfection.

### RNA interference

GSTM2 was widely expressed in various tissues including embryos, and testis with relative high expression^11^. Hence, GSTM2 could be expressed by normal ST cells. Three pairs of *GSTM2*-specific siRNAs were designed targeting corresponding regions of porcine *GSTM2* mRNA (Ambion). In addition, scrambled siRNA (Ambion) was used as a negative control. Briefly, the RNA interference transfection was performed as follows. Cell suspensions were reverse transfected in triplicate with siRNA via LipofectamineTM 2000 (Invitrogen, Carlsbad, CA, USA) and Optimem-I reduced serum media (OPTI-MEM I, Invitrogen) with the siRNA pooled at equal amounts to a final concentration of 50 nM. Six hours after transfection (Supplementary Fig. S1), the medium was refreshed, and the cells were further incubated for another 24 h. Triplicate wells of non-transfected cells were also included. To evaluate the effects of *GSTM2* knock-down on cells and transfection efficiency, we assessed morphology and cell numbers using fluorescence microscopy. The other two siRNAs, negative control and mock control followed the same treatments. Three independent experiments were performed.

### RNA isolation and real-time PCR

Total RNA isolated from transfected and control cells were assessed for integrity using a BioAnalyzer 2100 (Agilent, Santa Clara, USA) and for concentration and purity by the NanoDropTM 1000 Spectrophotometer (NanoDrop Technologies, Wilmington, DE, USA). After washes with Phosphate Buffered Saline (PBS), cells of each well were homogenized with 1 ml TRIzol reagent (Invitrogen) according to the manufacturer’s protocol followed by modifications after re-dissolving the RNA. The RNA was dissolved in 100 μL RNase-free water by mixing up and down with a pipette and stored at −80 °C freezer.

Real-time PCR was performed using a Roche LightCycler 480 detection system (Roche, Switzerland) and the following PCR program: denaturation at 95 °C for 30 sec, amplification for 40 cycles at 95 °C for 5 s, annealing and extension at 58 °C for 20 s and 72 °C for 10 s. Primer sequences and expected product sizes are shown in Supplementary Table S1. Specific amplification for certain PCRs was assessed by melting curve. One negative control reaction in which cDNA template was replaced by water was performed to avoid potential contamination. The sample from each well was repeated three times, and the comparative Ct (△△Ct) value method was used for relative quantification. Four genes, eukaryotic translation elongation factor 1 alpha 1(*EEF1A1*), heat shock protein 90 kDa alpha (cytosolic), class B member 1 (*HSPCB*), glyceraldehyde 3-phosphate dehydrogenase (*GAPDH*), and beta actin (*ACTB*), were chosen as potential housekeeping genes based on their uniformly high expression levels across groups from the sequencing data. Expression levels of these genes were assessed and used to normalize target genes via geNorm software^61^. Expression levels were considered undetectable when the Ct value of the targeted gene exceeded 35.

### Western blotting

Western blotting was used to further validate the effect of RNAi on *GSTM2* before RNA-seq. Transfected cells were homogenized in 1 ml of 25 mM Tris/1 mM ethylenediaminetetraacetic acid pH buffer, pH = 7.5. Homogenates were separated by 12.5% sodium dodecyl sulfate-polyacrylamide gel electrophoresis gels (SDS-PAGE) and transferred to a PVDF membrane (Millipore, Bedford, MA) using a semidry electrophoretic apparatus. The blocked membranes (5% BSA in TBS buffer containing 0.1% Tween 20) were incubated with anti-GSTM2 (1:1,000; AbCam, Cambridge, MA) and anti-beta-actin antibodies (1:1,000; AbCam, Cambridge, MA) overnight at 4 °C. The blots were extensively washed three times with TBST buffer for 10 min and incubated under gentle agitation with the secondary antibodies for immunodetection. The antigen-antibody reaction was incubated for 1 h, and the cross-reacting proteins were detected. Prestained molecular weight markers 10–170 kD in weight (Fermentas, Canada) were used as standards.

### RNA-seq

Deep sequencing was carried out at BGI (Shenzhen, China). Briefly, the total RNA samples were first treated with DNase I to degrade any possible contaminating DNA. Then, the mRNA was enriched using oligo (dT) magnetic beads (for eukaryotes) or by removing rRNAs from the total RNA (for prokaryotes). Mixed with the fragmentation buffer, the mRNA was fragmented into short fragments (approximately 200 bp). Then, the first strand of cDNA was synthesized using random hexamer primers. Buffer, dNTPs, RNase H and DNA polymerase I were added to synthesize the second strand. The double-stranded cDNA was purified with magnetic beads. End reparation and 3′-end single nucleotide A (adenine) addition was then performed. Finally, sequencing adaptors were ligated to the fragments, which were then enriched by PCR amplification. During the QC step, the Agilent 2100 Bioanaylzer and ABI StepOnePlus Real-Time PCR System were used to qualify and quantify the sample library. Total six cDNA libraries were constructed including three GSTM2-knock down groups and three negative control groups. The library products were sequenced using an Illumina HiSeqTM 2000. The RNA used in sequencing was the same with the samples for q-PCR analysis.

### Differential gene expression analysis

Values of RPKM were used to evaluate the total number of genes expression in each well of ST cells sample and the DEGs among each comparison^62^. The DEGs were analyzed based on an algorithm as previously described^62^. The P-value corresponds to a differential gene expression test in which False Discovery Rate (FDR) was used to determine the threshold of the P-value in multiple tests. The Cluster 3.0^63^ was used to the clustering analysis. The R heatmap package^64^ was applied to the analysis of Pearson and Spearman clustering. The functional classification of genes was performed using KEGG^65^ pathway analysis.

### Bioinformatics and data analysis

Identification of both pathway network and gene ontology (GO) categories was performed using IPA and the online tool PANTHER. All of the probe sequences from differential and positively expressed probes were first re-annotated with pig RefSeq RNA database from the porcine genome (Sscrofa10.2) from NCBI (Index of ftp://ftp.ncbi.nih.gov/genomes/Sus_scrofa/RNA/, last updated on October 2011). The unique gene symbol list from differential and positively detected probes was then uploaded into PANTHER (http://www.pantherdb.org/). The genes, transcripts, and proteins related to the Gene Ontology (GO) terms were identified. Then biological processes and pathways was obtained by these GO terms. The *Sus scrofa* genome was used as the reference gene list, which allowed for the identification of statistically significant biological processes and pathways from GO terms, which are represented in the over- and under-expressed between gene lists. More details related to the expected value and P-value calculation can be obtained online under Binomial Statistic Help from PANTHER.

### Co-immunoprecipitation assay

ST cells were plated in 100 mm dishes the day before transfection. Then cells were transfected with GFP-pcDNA3.1 containing the cDNA encoding GSTM2 and incubated at 37 °C in a humidified atmosphere of 5% CO_2_ for 24 h for transient expression. The GFP was used to confirm the equal amount of plasmid transfected each dish. The cells were washed with cold PBS three times and lysed in 1000 mL cell lysis buffer (Sangon Biotech, Shanghai). The lysate was centrifuged at 12,000 rpm for 5 min at 4 °C. The supernatant was divided into two equal parts, one added with 2 μg anti-GSTM2, the other added with 2 μL IgG as control, whereafter incubated at 4 °C for 5 h. Each aliquot was added with 50 μL Protein A/G beads (Millipore, USA) and incubated for 30 min. The beads were collected and rinsed with 1 ml PBS (containing 0.05% Tween 20) for 5 times (5 min one time). The beads were added with 20 μL water and 10 μL 5 × SDS PAGE buffer, boiled at 98 °C for 10 min and laid on ice for 5 min. The supernatant was saved for western blotting analysis.

### Overexpression and interference experiments

Complete CDS sequences of *GSTM2* and *STAT1* were obtained from NCBI. The Primers with restriction sites for full-length amplification were designed by Primer 5 software. Primer pairs were as follows: forward primer for *GSTM2* (5′-GGGGTACCCCGCGAGCAGGTCAGGGGAGAA-3′, underlined sequences recognized by *Kpn1*), reverse primer for *GSTM2* (5′-GCTCTAGAGCCATCTCCTGCTTCCAGGGCA-3′, underlined sequences recognized by *Xba1*), forward primer for *STAT1* (5′-CGGGATCCCGATGTCCCAGTGGTATGAGCTTC-3′, underlined sequences recognized by *BamH1*), reverse primer for *STAT1* (5′-GCTCTAGAGCTTAGTCAAGGTTCATAGTTCCAGAG-3′, underlined sequences recognized by *Xba1*). The amplification products were obtained by PCR with reaction mixture including 2 × Taq Master Mix 25 μL, cDNA library of ST cells 1 μL, forward primer 1 μL, reverse primer 1 μL, and dd water added to 50  μL. Then the PCR product was recovered by E.Z.N.A Gel Extraction Kit according to instructions (OMEGA), and additionally, double enzyme digestion reaction was performed with both PCR product and pcDNA3.1. The expression vectors of GSTM2 and STAT1 were acquired after connectivity, transformation, identification and DNA sequencing analysis. The expression vector of *GSTM2* or *STAT1* was transfected into ST cells followed by the same method as RNA interference experiment which was described previous, expect for the dose of expression vector for transfection was 4 μg (Supplementary Figs S5,S6).

### Statistical analyses

All experiments were repeated three times. Data were given as mean ± SD. Student’s t-test was used for statistical comparisons. P value < 0.05 was considered to be statistically significant.

## Additional Information

**How to cite this article**: Lv, Y. *et al*. Deep sequencing of transcriptome profiling of *GSTM2* knock-down in swine testis cells. *Sci. Rep.* **6**, 38254; doi: 10.1038/srep38254 (2016).

**Publisher’s note:** Springer Nature remains neutral with regard to jurisdictional claims in published maps and institutional affiliations.

## Supplementary Material

Supplementary Information
