# Supplementary material for: Deep sequencing of transcriptome profiling of GSTM2 knock-down in swine testis cells
Source: Sci Rep. 2016 Dec 1;6:38254. doi: 10.1038/srep38254 (PMC5131268; doi:10.1038/srep38254)
Supplement: Supplementary Information [file srep38254-s1.pdf]

Deep sequencing of transcriptome profiling of *GSTM2* knock-down in swine testis cells

Yuqi Lv[1, +], Yi Jin[1, +], Yongqiang Zhou[1], Jianjun Jin[1], Zhenfa Ma[1], Zhuqing Ren[1, 2,*]

[1]Key Laboratory of Swine Genetics and Breeding of Ministry of Agriculture & Key Laboratory of Agriculture Animal Genetics, Breeding and Reproduction of Ministry of Education, College of Animal Science, Huazhong Agricultural University, Wuhan, Hubei, P.R.China, 430070
[2]The Cooperative Innovation Center for Sustainable Pig Production, Huazhong Agricultural University, Wuhan, Hubei, P.R.China, 430070

[*]Corresponding author: renzq@mail.hzau.edu.cn (Zhuqing Ren)
[+]These authors contributed equally to this work

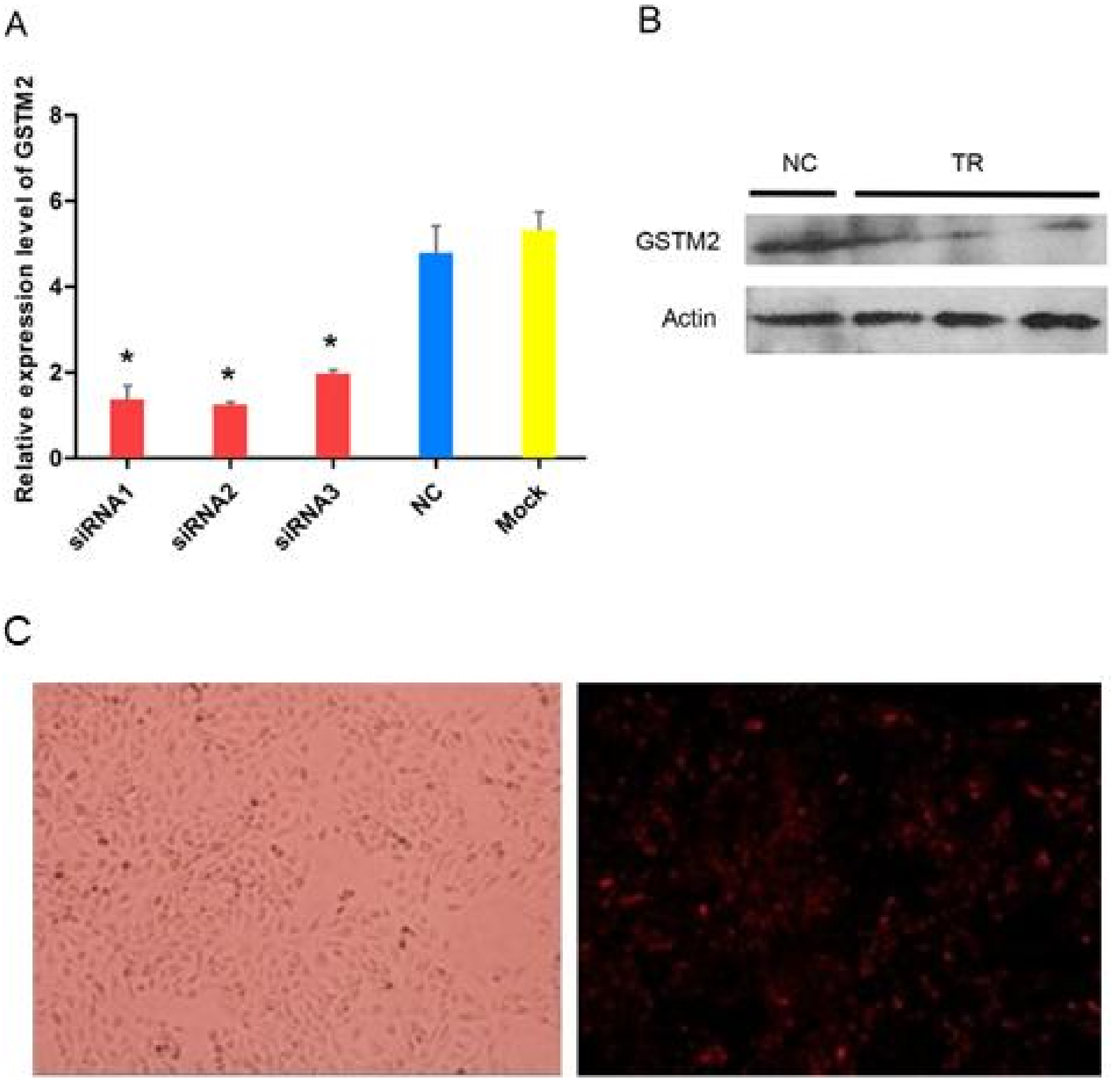

Supplementary Figure S1. Knock down *GSTM2* in ST cells transfected with siRNAs. A relative expression level of *GSTM2*. B. expression level of *GSTM2* by western blot analysis. C. detection of efficiency of siRNA transfection by fluorescence microscope. β-actin was used as the negative control. * $P < 0.05$, ** $P < 0.01$.

A

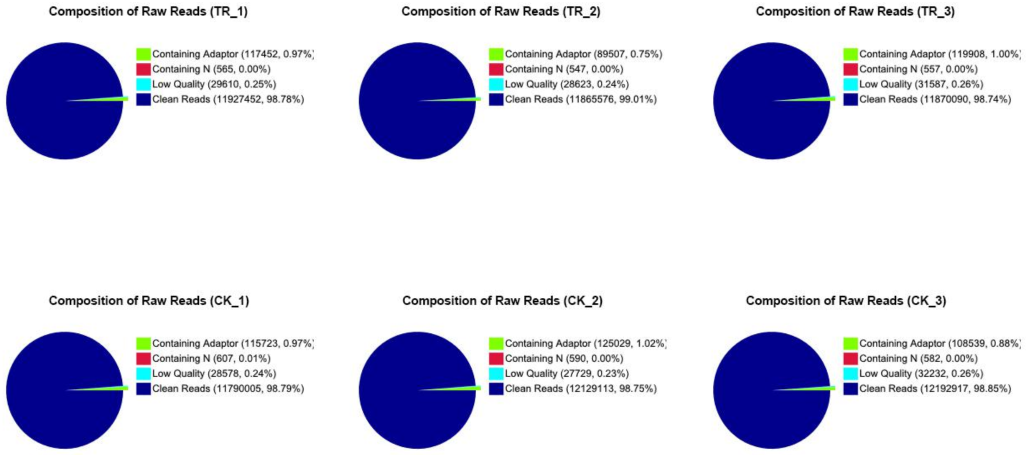
Composition of Raw Reads (TR_1)
Containing Adaptor (117452, 0.97%)
Containing N (565, 0.00%)
Low Quality (29610, 0.25%)
Clean Reads (11927452, 98.78%)
Composition of Raw Reads (TR_2)
Containing Adaptor (89507, 0.75%)
Containing N (547, 0.00%)
Low Quality (28623, 0.24%)
Clean Reads (11865576, 99.01%)
Composition of Raw Reads (TR_3)
Containing Adaptor (119908, 1.00%)
Containing N (557, 0.00%)
Low Quality (31587, 0.26%)
Clean Reads (11870090, 98.74%)
Composition of Raw Reads (CK_1)
Containing Adaptor (115723, 0.97%)
Containing N (607, 0.01%)
Low Quality (28578, 0.24%)
Clean Reads (11790005, 98.79%)
Composition of Raw Reads (CK_2)
Containing Adaptor (125029, 1.02%)
Containing N (590, 0.00%)
Low Quality (27729, 0.23%)
Clean Reads (12129113, 98.75%)
Composition of Raw Reads (CK_3)
Containing Adaptor (108539, 0.88%)
Containing N (582, 0.00%)
Low Quality (32232, 0.26%)
Clean Reads (12192917, 98.85%)

B

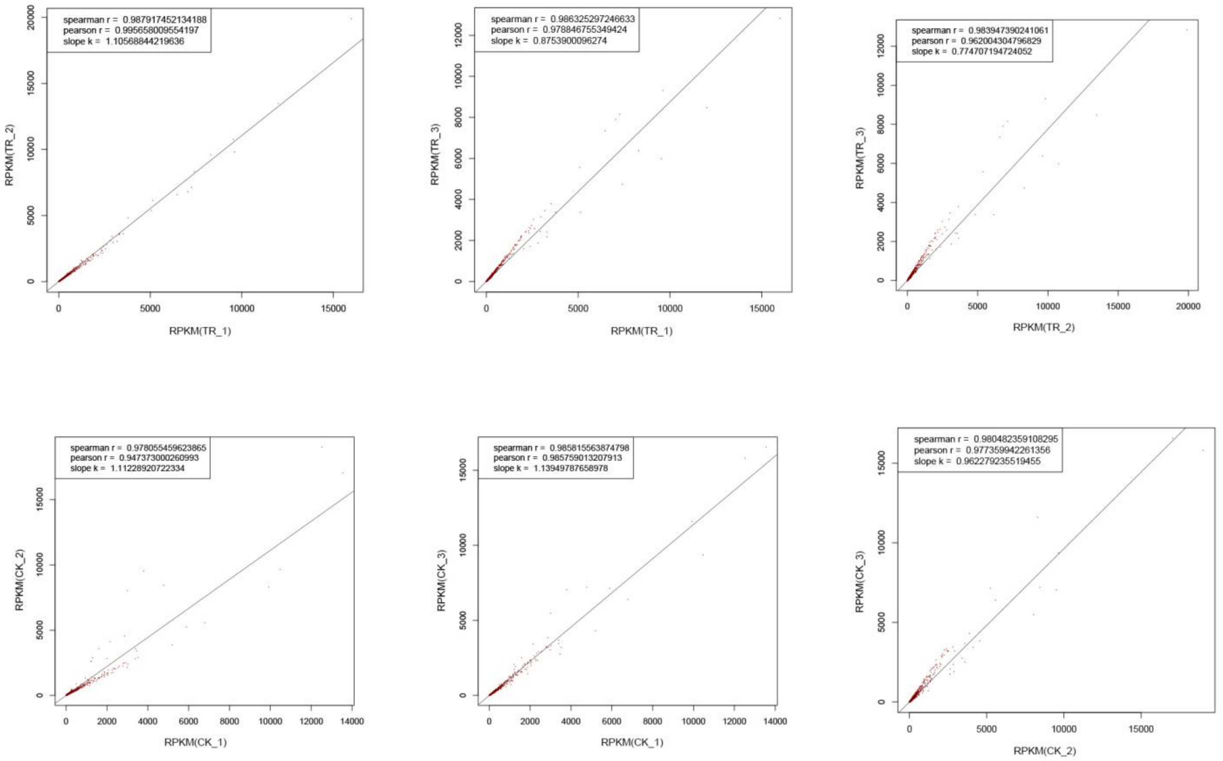
spearman r = 0.987917452134188
pearson r = 0.995658009554197
slope k = 1.10568844219636
RPKM(TR_1)
RPKM(TR_2)
spearman r = 0.986325297246633
pearson r = 0.978846755349424
slope k = 0.8753900096274
RPKM(TR_1)
RPKM(TR_3)
spearman r = 0.983947390241061
pearson r = 0.962004304796829
slope k = 0.774707194724052
RPKM(TR_2)
RPKM(TR_3)
spearman r = 0.978055459623865
pearson r = 0.947373000260993
slope k = 1.11228920722334
RPKM(CK_1)
RPKM(CK_2)
spearman r = 0.985815563874798
pearson r = 0.985759013207913
slope k = 1.13949787658978
RPKM(CK_1)
RPKM(CK_3)
spearman r = 0.980482359108295
pearson r = 0.977359942261356
slope k = 0.962279235519455
RPKM(CK_2)
RPKM(CK_3)

C

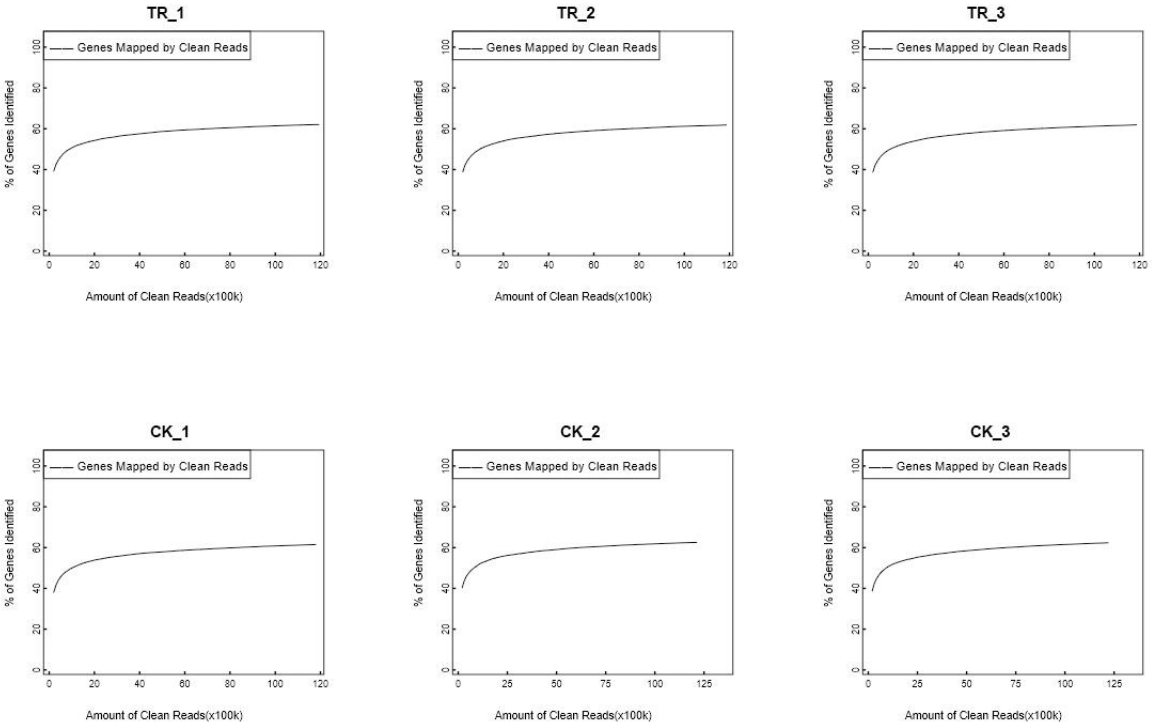

Supplementary Figure S2. Sequence quality and saturation analysis. A Total tag pie chart and distinct tag pie chart. The first row represents treatment group and the second row represents control group. The sample RNA preparation and experiments were thought to be convincing if the tags contains N were less than 10% of total raw data, and copy number less than 2 tags was no more than 20%. The data showed that all the samples conformed to sequencing requirement and the experiments were successful. B repeatability of RNA-seq. R values both from Spearman and Pearson analyses over 8 showed a high repeatability between two samples. C The saturation analysis. Sequence saturation analysis is used to measure the sequencing data of a sample. With the number of reads increasing, the number of detected genes is increasing. However, when the number of reads reaches a certain amount, the growth curve of detected genes flattens, which indicates that the number of detected genes tends to saturation.

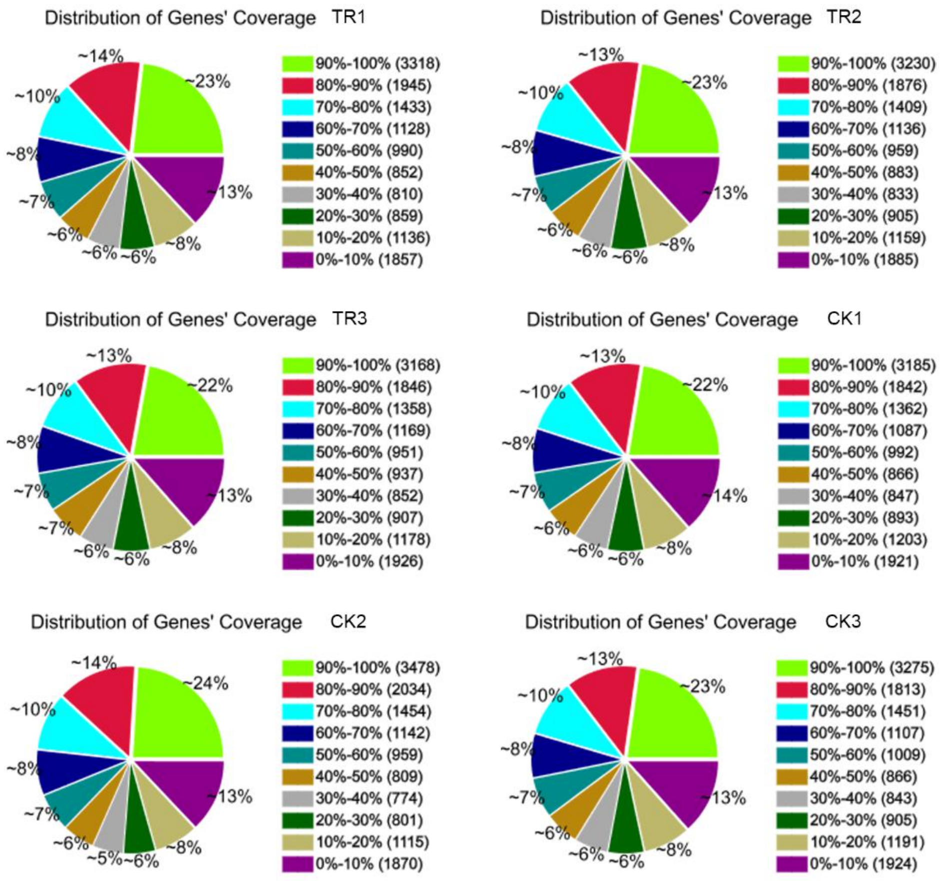

Supplementary Figure S3. Distribution of read coverage in transcripts investigated. (a) TR1. (b)TR2. (c) TR3. (d) CK1. (e) CK2. (f) CK3.

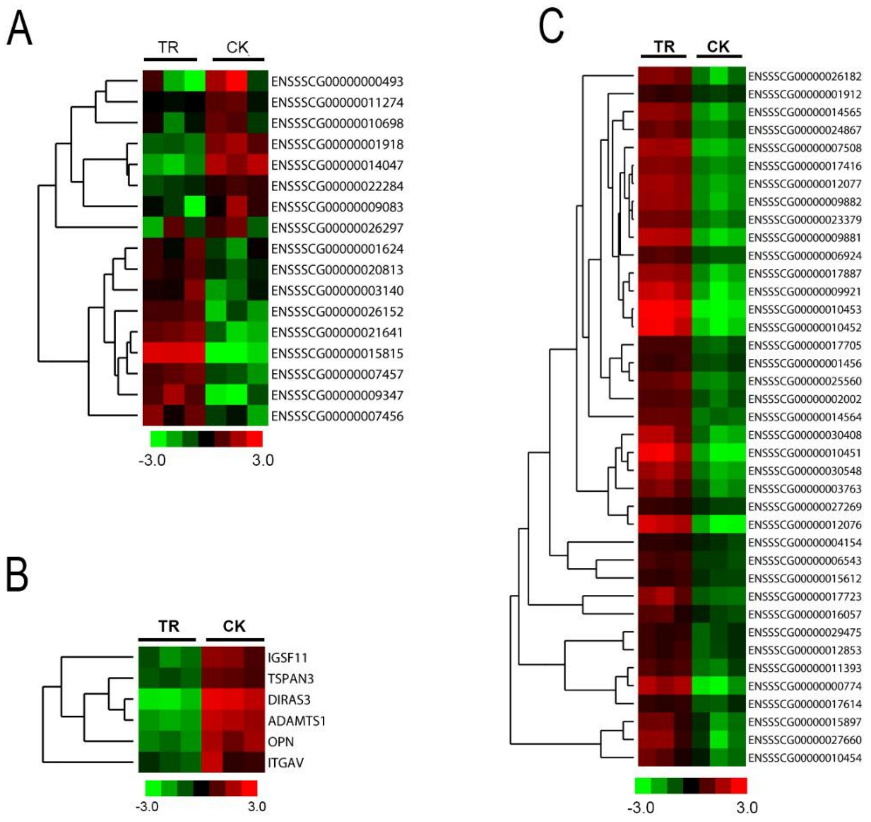

Supplementary Figure S4. Genes involved in the Maternal-Placental Interface and embryonic development. Each column indicates treatment groups (TR_1, TR_2, and TR_3) and negative control group. And each row means a specific gene. Expression differences are shown in different colors which red indicates up-regulated and green means down-regulated. A indicates cluster analysis of differential gene expression pattern in fibroblast growth factor receptor (*FGFR*) family. B indicates cluster analysis of differential gene expression pattern in cell adhesion (interface) process. C indicates cluster analysis of differential gene expression pattern in IFN family.

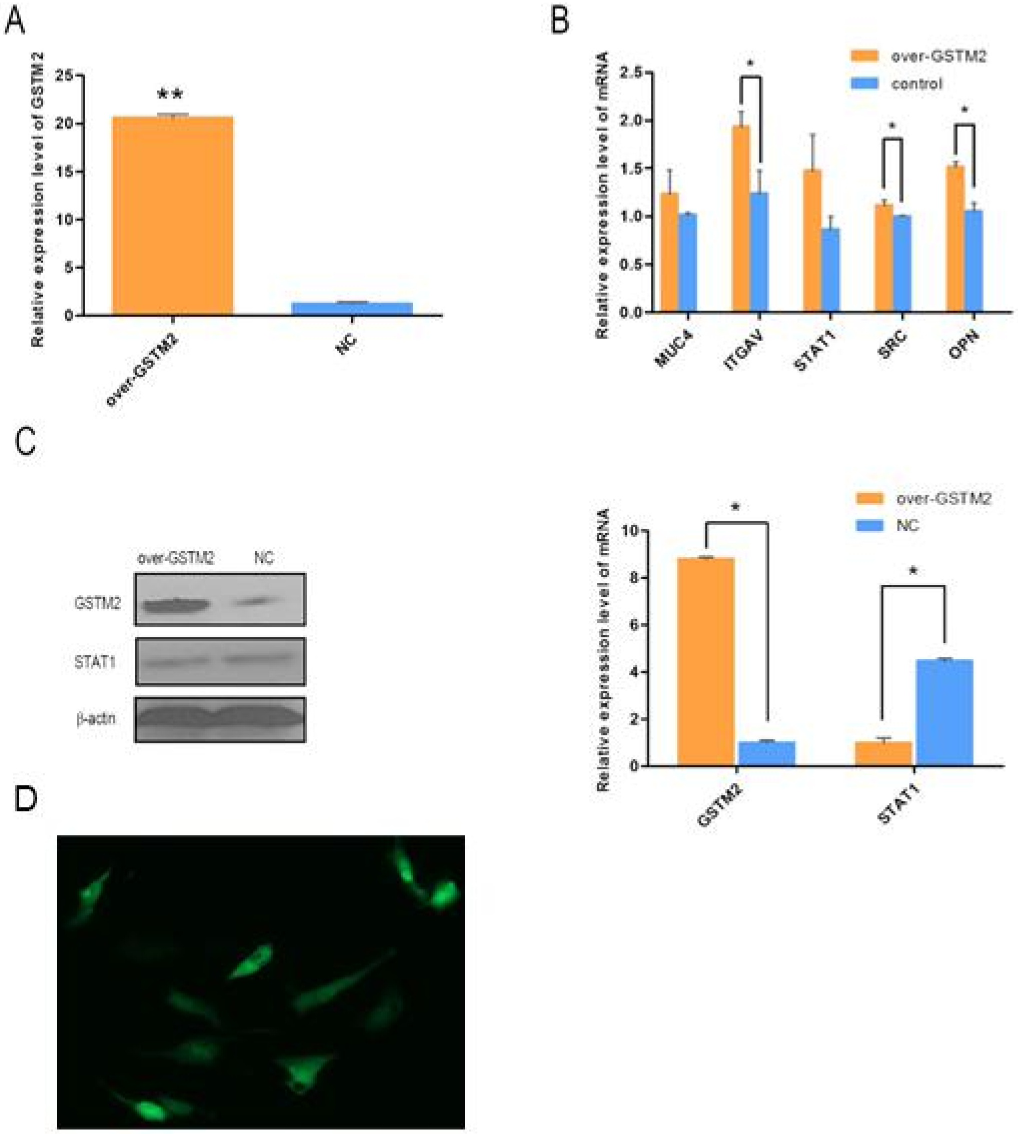

Supplementary Figure S5. Overexpression experiment of GSTM2. A *GSTM2* was overexpressed in ST cells and the mRNA expression level was detected after 48h. B downstream genes *MUC4, ITGAV, STAT1, SRC, OPN* mRNA expression level was tested after overexpressed *GSTM2* using q-PCR. C protein expression level of GSTM2 and STAT1 was investigated using western blot. The right pattern is the quantification of western blot of left pattern. D detection of transfection efficiency in ST cells by fluorescence microscope (40×).

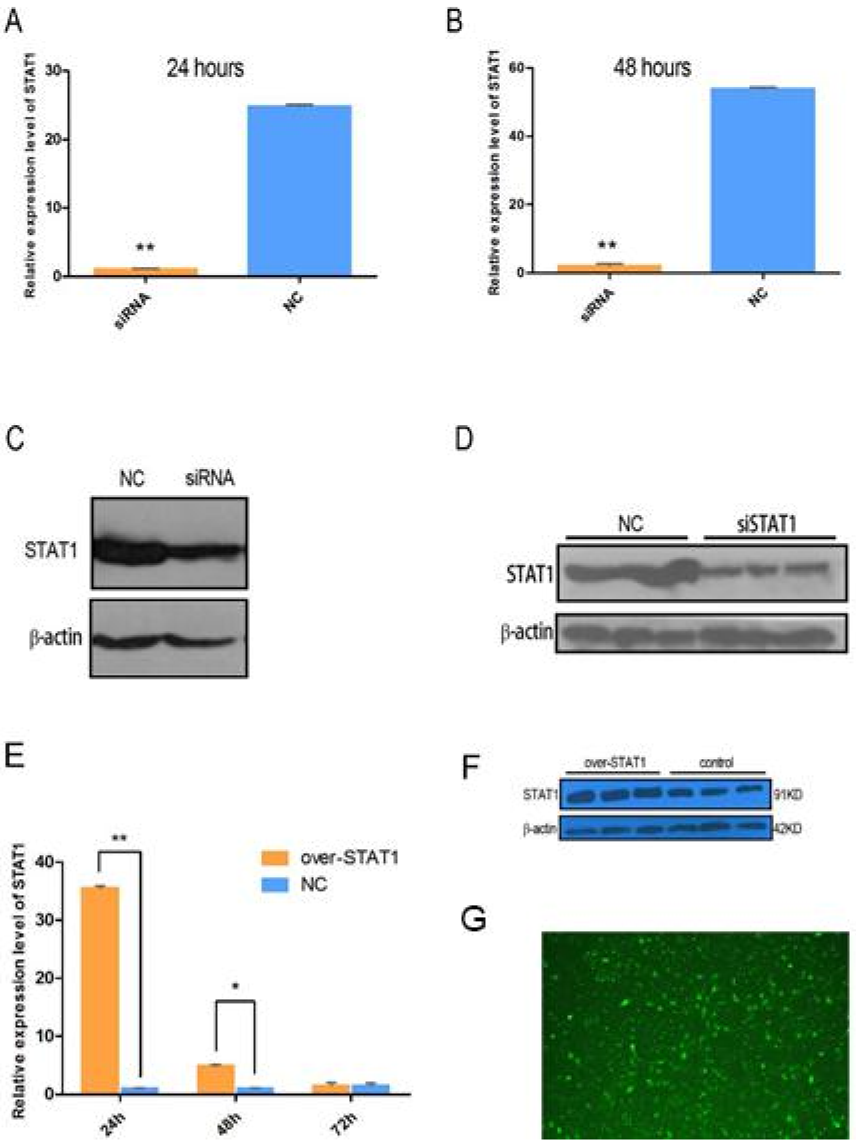

Supplementary Figure S6. Overexpression and interference experiment for *STAT1*. A-D Knock down *STAT1* in ST cells transfected with siRNA. A. the detection of *STAT1* mRNA level at 24h after transfection. B. the detection of STAT1 mRNA level at 48h after transfection. C the detection of STAT1 protein level at 24h after transfection. D the detection of STAT1 protein level at 48h after transfection. E-F. Overexpress *STAT1* in ST cells. The q-PCR and western blot were used to detect the mRNA and protein expression level in ST cells transfected with *STAT1* overexpression vector. E STAT1 mRNA expression level was increased in ST cells transfected with *STAT1* overexpression vector. F STAT1 protein expression level was increased in ST cells. G detection of transfection efficiency of siRNA. β-actin was used as the negative control. * $P < 0.05$, ** $P<0.01$.

Supplementary Table S1. Primers for real-time qPCR.

Gene symbol	Primer Sequence 5'-3'	Product size (bp)	Accession
SPP1	Forward: GAC AGC AGG GAC ACG GAC T<br>Reverse: TGC GGA ACT TCT TAG ATT TTG AC	220	NM_214023.1
STAT1	Forward: GGA ATG GAA GCG GAG ACA<br>Reverse: GGA AGA GGC TGA AGG TGC	218	NM_213769.1
ITGAV	Forward: ATG GGT TTG AGG TCT TCG<br>Reverse: CTT GTC TAT GTC CGT GGC	254	NM_001083932.1
EEF1A1	Forward: GTG AAA GCC ATC GCT AAA AG<br>Reverse: GTC CAA GAC CCA GGC ATA CT	213	NM_001097418.2
CYR61	Forward: AGG ACG GTG CCA AGG AC<br>Reverse: CGA GCA GAG GAG GGG TGT	186	XM_001927740.3
ADAMTS1	Forward: CAA AAG CAG CAC AAC CCG<br>Reverse: CAT CCG CCA TCC CAA GAG	118	NM_001144843.1
TIMP3	Forward: CGT GTC TAT GAT GGC AAG ATG TA<br>Reverse: AAG GCA GGT AGT AGC AGG ATT T	142	XM_003126073.4
DUSP1	Forward: ACA ACC ACA AGG CGG ACA TC<br>Reverse: TGC TCC TCC TCT GCT TCA CA	201	NM_001256075.1
MMP19	Forward: GCT GCC AGT GTC AGG TCA A<br>Reverse: ATG CGG AAG GTC AGG TGC	143	
MUC4	Forward: CAG TTT GTG AGA AGG ACG GT<br>Reverse: GAG GGG AGG GTT GGA GTA G	165	NM_001206344.1
SRC	Forward: GCA CTC ACT CAG CAC AGG ACA<br>Reverse: TCG TGG TTT CGC TTT CTC G	177	
IRG6	Forward: GCT GAA GGA AGC GGG TAT G<br>Reverse: GTT GCT CAC GAT GCT GAC ACT	139	KC109004.1
GAPDH	Forward: CGT CCC TGA GAC ACG ATG G<br>Reverse: TTC TCC GCC TTG ACT GTG C	200	NM_001206359
HSPCB	Forward: CGC TAC CAT ACC TCC CAG TC<br>Reverse: ACC AGG CTC TTC CCA TCA AA	236	
ACTB	Forward: AAG ATC AAG ATC ATC GCG CCT CCA<br>Reverse: ACT CCT GCT TGC TGA TCC ACA TCT	109	XM_003124280

Supplementary Table S2. Quality evaluation of transcriptome library. (a) Statistics of transcriptome library in each sample. (b) The number of reads mapping to reference gene and reference genome from each sample.

Sample	Raw reads	Clean reads
TR1	12,075,079	11,927,452 (98.78%)
TR2	11,984,253	11,865,576 (99.01%)
TR3	12,022,142	11,870,090 (98.74%)
CK1	11,934,913	11,790,005 (98.79%)
CK2	12,282,461	12,129,113 (98.75%)
CK3	12,334,270	12,192,917 (98.85%)

(a)

Sample	Total reads	Total BasePairs	Map to Gene		Map to Genome	
			Total mapped	Unique mapped	Total mapped	Unique mapped
TR1	11,927,452	584,445,148	7,766,274(65.11%)	6,134,237(51.43%)	8,876,534(74.42%)	7,428,666(62.28%)
TR2	11,865,576	581,413,224	7,498,252(63.19%)	5,930,876(49.98%)	8,767,986(73.89%)	7,308,617(61.60%)
TR3	11,870,090	581,634,410	7,702,473(64.89%)	6,046,769(50.94%)	8,749,773(73.71%)	7,297,390(61.48%)
CK1	11,790,005	577,710,245	8,069,266(68.44%)	6,415,439(54.41%)	8,930,091(75.74%)	7,480,412(63.45%)
CK2	12,129,113	594,326,537	8,188,041(67.51%)	6,562,121(54.10%)	9,281,992(76.53%)	7,815,559(64.44%)
CK3	12,192,917	597,452,93	8,301,723(68.09%)	6,605,101(54.17%)	9,237,580(75.76%)	7,753,997(63.59%)

(b)

Supplementary Table S3. The reads mapping to reference gene and reference genome from TR and CK groups. (a) TR1. (b) TR2. (c) TR3. (d) CK1. (e) CK2. (f) CK3.

TR1	Map to gene		Map to genome	
	Reads number	percentage	Reads number	percentage
Total Reads	11927452	100.00%	11927452	100.00%
Total BasePairs	584445148	100.00%	584445148	100.00%
Total Mapped Reads	7766274	65.11%	8876534	74.42%
perfect match	6433722	53.94%	7003970	58.72%
<=2bp mismatch	1332552	11.17%	1872564	15.70%
unique match	6134237	51.43%	7428666	62.28%
multi-position match	1632037	13.68%	1447868	12.14%
Total Unmapped Reads	4161178	34.89%	3050918	25.58%

(a)

TR2	Map to gene		Map to genome	
	Reads number	percentage	Reads number	percentage
Total Reads	11865576	100.00%	11865576	100.00%
Total BasePairs	581413224	100.00%	581413224	100.00%
Total Mapped Reads	7498252	63.19%	8767986	73.89%
perfect match	6214289	52.37%	6924523	58.36%
<=2bp mismatch	1283963	10.82%	1843463	15.54%
unique match	5930876	49.98%	7308617	61.60%
multi-position match	1567376	13.21%	1459369	12.30%
Total Unmapped Reads	4367324	36.81%	3097590	26.11%

(b)

TR3	Map to gene		Map to genome	
	Reads number	percentage	Reads number	percentage
Total Reads	11870090	100.00%	11870090	100.00%
Total BasePairs	581634410	100.00%	581634410	100.00%
Total Mapped Reads	7702473	64.89%	8749773	73.71%
perfect match	6355147	53.54%	6866078	57.84%
<=2bp mismatch	1347326	11.35%	1883695	15.87%
unique match	6046769	50.94%	7297390	61.48%
multi-position match	1655704	13.95%	1452383	12.24%
Total Unmapped Reads	4167617	35.11%	3120317	26.29%

(c)

CK1	Map to gene		Map to genome	
	Reads number	percentage	Reads number	percentage
Total Reads	11790005	100.00%	11790005	100.00%
Total BasePairs	577710245	100.00%	577710245	100.00%

Total Mapped Reads	8069266	68.44%	8930091	75.74%
perfect match	6713511	56.94%	7030307	59.63%
<=2bp mismatch	1355755	11.50%	1899784	16.11%
unique match	6415439	54.41%	7480412	63.45%
multi-position match	1653827	14.03%	1449679	12.30%
Total Unmapped Reads	3720739	31.56%	2859914	24.26%

(d)

CK2	Map to gene		Map to genome	
	Reads number	percentage	Reads number	percentage
Total Reads	12129113	100.00%	12129113	100.00%
Total BasePairs	594326537	100.00%	594326537	100.00%
Total Mapped Reads	8188041	67.51%	9281992	76.53%
perfect match	6860359	56.56%	7380066	60.85%
<=2bp mismatch	1327682	10.95%	1901926	15.68%
unique match	6562121	54.10%	7815559	64.44%
multi-position match	1625920	13.41%	1466433	12.09%
Total Unmapped Reads	3941072	32.49%	2847121	23.47%

(e)

CK3	Map to gene		Map to genome	
	Reads number	percentage	Reads number	percentage
Total Reads	12192917	100.00%	12192917	100.00%
Total BasePairs	597452933	100.00%	597452933	100.00%
Total Mapped Reads	8301723	68.09%	9237580	75.76%
perfect match	6894131	56.54%	7247842	59.44%
<=2bp mismatch	1407592	11.54%	1989738	16.32%
unique match	6605101	54.17%	7753997	63.59%
multi-position match	1696622	13.91%	1483583	12.17%
Total Unmapped Reads	3891194	31.91%	2955337	24.24%

(f)

Supplementary Table S4. The differentially expressed genes in treatment groups compared to control groups.

ENSEMBL_ID	GENE_SYMBOL	GB_ACC	log2Ratio(TR/CK)	FDR
ENSSSCG00000010453	IFIT1	NM_001244363	6.361719332	0
ENSSSCG00000008648	IRG6	NM_213817	5.647394281	0
ENSSSCG00000010452	IFIT3	NM_001204395	5.557832768	0
ENSSSCG00000010451	IFIT2	5409	5.353464687	7.84E-45
ENSSSCG00000012076	MX2	NP_001090885	5.170004159	0
ENSSSCG00000009921	OASL	NM_001031790	4.775356695	0
ENSSSCG00000000774	USP18	NM_213826	4.63895955	7.05E-46
ENSSSCG00000004192	CTGF	NP_998998	4.637635534	1.90E-133
ENSSSCG00000009720	DDX60	25942	4.609178464	0
ENSSSCG00000009881	OAS2	NM_001031796	4.502464045	0
ENSSSCG00000021766	novel		4.34866761	1.81E-34
ENSSSCG00000027982	ISG15	NM_001128469	4.196848933	0
ENSSSCG00000021712	HERC6	26072	4.137676877	4.65E-195
ENSSSCG00000007508	ZBP1	NM_001123216	3.993848525	3.01E-121
ENSSSCG00000030408	DDX58	NM_213804	3.982813341	1.43E-134
ENSSSCG00000017887	XAF1	30932	3.817863407	4.57E-219
ENSSSCG00000012077	MX1	NM_214061	3.660580296	5.77E-221
ENSSSCG00000030548	HERC5	24368	3.61425212	2.99E-196
ENSSSCG00000009882	OAS1	F1RJN6	3.613993059	4.22E-102
ENSSSCG00000008647	CMPK2	27015	3.566528178	0
ENSSSCG00000014565	novel	F1RGC4	3.478675612	3.84E-114

ENSSSCG00000000148	novel	F1SPU1	3.415696751	2.15E-268
ENSSSCG00000011936	ZBED2	20710	3.302139681	0
ENSSSCG00000027372	SAMD9	1348	3.293354012	5.48E-249
ENSSSCG00000006590	S100A8	NP_001153743	3.173914301	1.26E-188
ENSSSCG00000026182	novel	I3LK92	3.124554811	1.01E-91
ENSSSCG00000017416	DHX58	NM_001199132	3.106373417	0
ENSSSCG00000024430	HES4	24149	3.087923901	1.28E-32
ENSSSCG00000027660	IFI44L	17817	3.029625761	2.25E-45
ENSSSCG00000002919	TYROBP	NM_214202	2.896003237	7.56E-18
ENSSSCG00000017723	CCL2	NM_214214	2.864923623	1.41E-25
ENSSSCG00000003763	IFI44	16938	2.807764245	1.81E-18
ENSSSCG00000023379	UBE2L6	F4ZS20	2.764804107	3.54E-15
ENSSSCG00000010461	ANKRD1	NM_213922	2.740148077	9.90E-285
ENSSSCG00000011874	PARP14	29232	2.697062015	1.50E-110
ENSSSCG00000017698	CCL4	NM_213779	2.634411825	9.22E-07
ENSSSCG00000025560	PGLYRP2	NM_213738	2.561872937	3.12E-112
ENSSSCG00000028525	SAA4	NM_001044552	2.555652415	1.23E-45
ENSSSCG00000006940	CYR61	F1SEX1	2.528078759	1.35E-31
ENSSSCG00000001394	SUSC-MIC1	NM_001114274	2.523276097	1.11E-298
ENSSSCG00000013940	NLRP3	16400	2.500425721	1.50E-37
ENSSSCG00000024867	ISG20	6130	2.497551336	4.53E-39
ENSSSCG00000002471	ISG12	NM_001198921	2.474902616	4.30E-18
ENSSSCG00000014564	IFITM1	A9QW81	2.473111131	2.06E-61
ENSSSCG00000015897	IFIH1		2.43203815	0

ENSSSCG00000016233	PN-1	NM_214287	2.399370672	1.66E-25
ENSSSCG00000006719	HSD3B1	Q9N119	2.374062474	6.32E-202
ENSSSCG00000000155	TIMP3	11822	2.327259507	1.01E-261
ENSSSCG00000010454	IFIT5	13328	2.316026639	1.38E-11
ENSSSCG00000026832	CSF1	NM_001244523	2.301142662	9.59E-09
ENSSSCG00000026174	GOLGA7B	31668	2.298618499	2.55E-59
ENSSSCG00000006815	novel		2.184748274	9.10E-41
ENSSSCG00000011393	UBA7	12471	2.168305627	4.08E-66
ENSSSCG00000016254	CCL20	NM_001024589	2.136009658	0
ENSSSCG00000013369	SAA1		2.116214736	2.76E-24
ENSSSCG00000005316	TPM2	NM_001129947	2.106762938	1.40E-06
ENSSSCG00000022447	F3	3541	2.04309922	9.24E-09
ENSSSCG00000016535	CALD1	1441	2.030235975	0
ENSSSCG00000002002	IRF9	6131	2.017247555	1.74E-131
ENSSSCG00000025206	RNF19B	26886	2.000412109	6.74E-99
ENSSSCG00000014780	TRIM21	11312	1.97280739	1.20E-34
ENSSSCG00000011876	DTX3L	30323	1.956583841	3.00E-64
ENSSSCG00000021943	novel	I3LPW3	1.935349714	1.84E-48
ENSSSCG00000006924	GBP1	NM_001128473	1.934150952	4.27E-59
ENSSSCG00000023178	BATF2	25163	1.884668381	5.06E-08
ENSSSCG00000017705	CCL5	NM_001129946	1.881750454	1.15E-05
ENSSSCG00000016262	SP140	17133	1.838525911	1.52E-68
ENSSSCG00000017146	RNF213	14539	1.83341397	3.22E-99
ENSSSCG00000001473	COL11A2	A5D9L2	1.749090861	1.18E-40

ENSSSCG00000016925	PLK2	19699	1.729104657	1.66E-181
ENSSSCG00000010312	PLAU	9052	1.721323486	2.78E-75
ENSSSCG00000017148	novel	F1RZA4	1.719165534	4.93E-220
ENSSSCG00000014670	TRIM5	NM_001044532	1.685084743	1.68E-40
ENSSSCG00000016057	STAT1	NM_213769	1.679772215	4.14E-19
ENSSSCG00000006543	ADAR	225	1.676152574	0.000296531
ENSSSCG00000006681	TXNIP	NM_001044614	1.668097705	5.88E-40
ENSSSCG00000005625	ENG	3349	1.619076045	1.14E-39
ENSSSCG00000016991	DUSP1	F1RS00	1.616680241	6.14E-39
ENSSSCG00000011904	UPK1B	12578	1.602779784	2.11E-79
ENSSSCG00000020893	TAP	NM_001044581	1.586974995	1.22E-197
ENSSSCG00000014072	ENC1	3345	1.571871148	3.67E-38
ENSSSCG00000026759	HMOX1	5013	1.570701244	3.83E-20
ENSSSCG00000001456	SLA-DQA1	NM_001130224	1.543092652	4.22E-24
ENSSSCG00000015073	TAGLN	11553	1.527239936	1.52E-28
ENSSSCG00000012853	IRF7	NM_001097428	1.525849128	1.20E-33
ENSSSCG00000028384	RND3	671	1.515058009	5.25E-90
ENSSSCG00000011702	TM4SF1	11853	1.492741628	1.33E-14
ENSSSCG00000029475	novel	I3LLF7	1.485275207	2.06E-28
ENSSSCG00000017614	TRIM25	12932	1.455396743	0
ENSSSCG00000017105	UBE2QL1	37269	1.438739483	5.08E-93
ENSSSCG00000008953	IL8	NM_213867	1.436370049	3.78E-65
ENSSSCG00000013599	ANGPTL4	NM_001038644	1.399484763	1.50E-10
ENSSSCG00000022913	SLPI	11092	1.364977709	1.50E-192

ENSSSCG00000001912	PML	9113	1.356580251	2.69E-29
ENSSSCG00000004682	B2M	NM_213978	1.350040322	6.01E-233
ENSSSCG00000027269	novel		1.333978742	1.27E-113
ENSSSCG00000022484	CH242-228L8.2	F1RTC7	1.332106997	5.91E-126
ENSSSCG00000021359	novel	F1S4Q9	1.32774076	6.37E-161
ENSSSCG00000015612	IRF6	NM_214278	1.29744568	5.50E-22
ENSSSCG00000017087	GM2A	4367	1.288064776	1.56E-34
ENSSSCG00000007032	PLAT	9051	1.280079963	4.39E-33
ENSSSCG00000006589	S100A12	NM_001160272	1.272862434	9.76E-28
ENSSSCG00000000368	MMP19	7165	1.261609326	1.36E-42
ENSSSCG00000022111	novel	I3LK35	1.254390066	9.85E-13
ENSSSCG00000024161	SLA-7	NM_213768	1.251768684	6.42E-63
ENSSSCG00000012890	TCIRG1	11647	1.247861397	1.98E-80
ENSSSCG00000001233	TRIM26	NM_001123209	1.246565759	6.52E-47
ENSSSCG00000029949	CD248	18219	1.235130515	7.68E-12
ENSSSCG00000017616	novel	F1RSC4	1.214379101	3.23E-27
ENSSSCG00000003451	PDPN	29602	1.211525369	8.19E-128
ENSSSCG00000027607	IER3	5392	1.175048265	9.24E-26
ENSSSCG00000011357	SHISA5	30376	1.151323247	5.15E-215
ENSSSCG00000001341	SLA-11	F1RUD0	1.140348803	1.34E-98
ENSSSCG00000004154	TNFAIP3	11896	1.131683804	2.01E-23
ENSSSCG00000003832	TACSTD2	11530	1.126387196	6.09E-27
ENSSSCG00000017082	novel	F1RQB3	1.110435362	1.37E-09
ENSSSCG00000017754	LGALS9	NM_213932	1.102162281	3.25E-61

ENSSSCG00000028304	ZFP36L1	1107	1.088003423	4.01E-81
ENSSSCG00000022576	VGF	12684	1.057135021	1.73E-66
ENSSSCG00000030835	BLCAP	1055	1.043885476	8.16E-75
ENSSSCG00000017199	TRIM47	19020	1.021622797	4.06E-54
ENSSSCG00000022370	TNFSF9	11939	1.01473239	5.71E-33
ENSSSCG00000026169	MFSD12	28299	1.01314651	0.000134909
ENSSSCG00000000293	ITGA5	F1SR53	1.013138853	2.10E-10
ENSSSCG00000010624	DUSP5	3071	1.00681917	1.01E-48
ENSSSCG00000021641	PDGFB	8800	1.003927499	9.01E-59
ENSSSCG00000016117	CARF	14435	1.003542798	1.22E-09
ENSSSCG00000001377	PPP1R18	29413	0.973670325	7.00E-32
ENSSSCG00000012873	CCND1	1582	0.969229042	3.17E-128
ENSSSCG00000024669	NBL1	I3LKM2	0.966556093	1.06E-15
ENSSSCG00000029998	KLF7	NP_001090956	0.950009297	1.66E-25
ENSSSCG00000002704	KARS	6215	-0.911171633	2.21E-153
ENSSSCG00000000849	HSP90B1	12028	-0.93452704	1.13E-39
ENSSSCG00000021332	PAX8	8622	-0.971018097	1.47E-115
ENSSSCG00000009978	AP1B1	554	-0.975159854	1.02E-31
ENSSSCG00000004963	novel	F1SIU2	-0.978779937	9.95E-27
ENSSSCG00000025437	novel	I3LVG0	-0.986758757	1.09E-62
ENSSSCG00000009501	DZIP1	20908	-0.992195201	2.72E-28
ENSSSCG00000007334	SRC	11283	-0.996333221	5.31E-196
ENSSSCG00000005934	TRAPPC9	30832	-1.008723934	1.81E-150
ENSSSCG00000010118	HIRA	4916	-1.02769447	1.46E-265

ENSSSCG00000000522	NAP1L1	NM_001244316	-1.029229206	4.58E-36
ENSSSCG00000027525	DHCR24	NM_001243354	-1.044194011	8.23E-15
ENSSSCG00000004058	EZR	12691	-1.052407347	2.03E-18
ENSSSCG00000028960	novel	I3L7C2	-1.055723347	0
ENSSSCG00000005663	CCBL1	1564	-1.057286121	1.39E-103
ENSSSCG00000028381	FLYWCH1	25404	-1.058790047	5.90E-06
ENSSSCG00000015983	HOXD8	5139	-1.062380214	1.69E-63
ENSSSCG00000025831	NREP	F1RLG1	-1.071542751	8.70E-53
ENSSSCG00000000094	SUN2	14210	-1.075663689	1.16E-136
ENSSSCG00000010554	SCD	NM_213781	-1.075951669	2.98E-35
ENSSSCG00000022858	CS	P00889	-1.076648505	3.88E-103
ENSSSCG00000005601	HSPA5	F1RS36	-1.083246933	6.61E-71
ENSSSCG00000020821	novel	I3LR79	-1.086014937	1.00E-10
ENSSSCG00000015105	SLC37A4	NM_001199719	-1.086474577	2.39E-69
ENSSSCG00000016974	MAP1B	6836	-1.087606455	8.58E-249
ENSSSCG00000012247	ATP6AP2	F1RXN8	-1.109353981	5.47E-58
ENSSSCG00000021091	CS	2422	-1.135976133	8.74E-25
ENSSSCG00000004206	novel	F1S2Z5	-1.148387078	8.83E-42
ENSSSCG00000023050	SEPT10	14349	-1.162699321	5.15E-156
ENSSSCG00000015849	TMEM66	28789	-1.167614071	1.06E-17
ENSSSCG00000024388	BNIP3	1084	-1.172427348	1.24E-32
ENSSSCG00000007160	DDRGK1	NM_001190212	-1.186934894	3.36E-24
ENSSSCG00000025941	PTP4A1	I3L887	-1.190094083	8.42E-50
ENSSSCG00000029698	LRRC42	28792	-1.191801907	1.31E-121

ENSSSCG00000003806	LEPROT	NM_001145388	-1.208367689	8.60E-35
ENSSSCG00000008733	RAB28	9768	-1.209695468	1.31E-14
ENSSSCG00000017666	CH242-205G24.7	NP_001139599	-1.216645729	6.14E-52
ENSSSCG00000027689	SRM	11296	-1.216726744	2.88E-29
ENSSSCG00000008466	COX7A2L	2289	-1.227413098	1.02E-30
ENSSSCG00000016975	PTCD2	25734	-1.228596555	1.62E-118
ENSSSCG00000000022	LDOC1L	13343	-1.2293588	7.00E-32
ENSSSCG00000016027	ITGAV	NM_001083932	-1.232109973	1.37E-114
ENSSSCG00000024752	ALDH4A1	406	-1.235066987	1.41E-12
ENSSSCG00000015103	RPS25	10413	-1.240711651	1.42E-77
ENSSSCG00000007692	YWHAG	12852	-1.251160315	2.40E-07
ENSSSCG00000015797	SORBS2	P28220	-1.264979592	2.71E-45
ENSSSCG00000014833	UCP2	NM_214289	-1.278954094	1.60E-24
ENSSSCG00000014047	FGFR4	F1S3B8	-1.280439152	1.21E-162
ENSSSCG00000008602	LAPTM4A	6924	-1.298883338	2.42E-16
ENSSSCG00000028727	novel	I3L7W2	-1.30093721	3.03E-32
ENSSSCG00000021104	novel		-1.304858391	2.92E-13
ENSSSCG00000011850	MUC4	NM_001206344	-1.306405144	6.56E-21
ENSSSCG00000027669	TNS1	11973	-1.315091853	3.58E-06
ENSSSCG00000001008	BPHL	1094	-1.326616455	1.26E-194
ENSSSCG00000027417	LDLRAD4	1224	-1.328713366	2.47E-112
ENSSSCG00000002718	FA2H	21197	-1.336913	9.86E-22
ENSSSCG00000012365	MSN	NM_001009578	-1.345821796	3.42E-53
ENSSSCG00000022659	CLTC	I3LGD4	-1.362579682	3.65E-17

ENSSSCG00000001868	TSPAN3	NM_001244196	-1.381873369	6.50E-19
ENSSSCG00000006544	UBE2Q1	15698	-1.390424111	3.50E-98
ENSSSCG00000017880	SPNS3	28433	-1.390938786	0
ENSSSCG00000008966	PARM1	NM_001243716	-1.397623568	1.93E-36
ENSSSCG00000030240	novel	I3LIP1	-1.40481458	8.55E-256
ENSSSCG00000011133	PFKFB3	8874	-1.411213043	2.37E-57
ENSSSCG00000002740	AP1G1	F1S3C7	-1.449088285	1.21E-52
ENSSSCG00000010543	ABCC2	53	-1.454793573	0
ENSSSCG00000022009	AADC	NP_999019	-1.456366914	3.45E-31
ENSSSCG00000012774	DUSP9		-1.461140011	2.52E-145
ENSSSCG00000008274	DCTN1	F1SNT8	-1.465024137	9.77E-06
ENSSSCG00000030648	novel	I3LPB3	-1.473844669	2.02E-51
ENSSSCG00000028069	ATP2B4	I3LSA3	-1.476953589	3.51E-17
ENSSSCG00000027256	CNIH	NM_001243525	-1.513032432	7.16E-46
ENSSSCG00000015273	novel	F1S6B3	-1.529242285	1.32E-64
ENSSSCG00000027450	novel	NP_001230531	-1.531083624	4.38E-30
ENSSSCG00000011906	IGSF11	16669	-1.538886858	6.29E-29
ENSSSCG00000016695	novel	F1SHU0	-1.543001725	8.64E-07
ENSSSCG00000001137	HIST1H1D	4717	-1.55593831	1.43E-55
ENSSSCG00000004586	FAM81A	28379	-1.584223556	5.23E-28
ENSSSCG00000011845	PCYT1A	8754	-1.606815184	0
ENSSSCG00000029589	novel	I3LUZ8	-1.613534595	2.73E-31
ENSSSCG00000017448	KRT19	F1S0J8	-1.614922506	4.84E-23
ENSSSCG00000014125	DHFR	NM_001244064	-1.670357601	1.09E-215

ENSSSCG00000006838	TMEM167B	F1S5Z0	-1.675569947	5.01E-54
ENSSSCG00000005491	ATP6V1G1	NM_001190194	-1.685455512	1.96E-171
ENSSSCG00000014073	HEXB	NM_213921	-1.706219402	1.66E-217
ENSSSCG00000016370	SEPT2	NM_001243793	-1.766021157	5.54E-245
ENSSSCG00000008449	SLC3A1	NM_001123042	-1.782251972	0
ENSSSCG00000022417	HNF1B	NM_213956	-1.793501374	0
ENSSSCG00000012957	SF3B2	F1RU38	-1.800687321	4.62E-85
ENSSSCG00000004807	SCG5	NP_001098757	-1.800742784	3.96E-29
ENSSSCG00000028740	CTDSPL	I3L819	-1.80138116	0
ENSSSCG00000017885	SMTNL2	F1RGN8	-1.957409172	5.21E-19
ENSSSCG00000021564	IRX5	I3L8M8	-2.001943613	1.25E-48
ENSSSCG00000003617	TXLNA		-2.160652899	2.85E-12
ENSSSCG00000009216	SPP1	NM_214023	-2.172777202	2.40E-10
ENSSSCG00000015798	ANKRD37	F1RZN8	-2.180607007	2.63E-87
ENSSSCG00000006693	PDZK1	8821	-2.2445841	4.00E-283
ENSSSCG00000005240	DOCK8	F1SJE9	-2.294181514	2.65E-06
ENSSSCG00000016872	HMGCS1	F1SMG8	-2.374787687	2.38E-05
ENSSSCG00000003744	novel	F1SAI5	-2.396718131	5.80E-21
ENSSSCG00000029488	ADAMTS1	I3LDG0	-2.415897687	4.11E-27
ENSSSCG00000000500	RAB3IP	F1SH42	-2.503477823	3.75E-263
ENSSSCG00000018048	SLC5A10	NM_001012297	-2.571092456	5.76E-17
ENSSSCG00000012026	ADAMTS1	NM_001144843	-2.572268516	2.04E-37
ENSSSCG00000010400	MSMB	NM_213852	-2.576722987	5.26E-95
ENSSSCG00000004920	novel	F1SM36	-3.016082575	2.52E-05

ENSSSCG00000004676	DUOXA2	NM_001243550	-3.277297188	2.91E-17
ENSSSCG00000024743	PRR15L	I3LNV1	-3.354929211	0.000456212
ENSSSCG00000003797	DIRAS3	NM_001044598	-3.495079167	8.51E-33
ENSSSCG00000005979	ANXA13	F1RRP6	-3.872277985	9.67E-77
ENSSSCG00000011663	RBP2	NM_214451	-9.067476454	4.87E-06

Supplementary Table S5. Gene Ontology analysis for DEGs. (a) Biological process. (b) Molecular function. (c) Cell component.

Items	fold Enrichment	P-value
ISG15-protein conjugation (GO:0032020)	67.93	2.91E-03
negative regulation of viral genome replication (GO:0045071)	21.08	3.40E-03
negative regulation of viral process (GO:0048525)	17.64	2.20E-05
negative regulation of viral life cycle (GO:1903901)	17.64	2.20E-05
neutrophil chemotaxis (GO:0030593)	14.56	2.82E-02
defense response to virus (GO:0051607)	13.99	1.96E-08
regulation of viral genome replication (GO:0045069)	13.29	4.71E-02
neutrophil migration (GO:1990266)	13.29	4.71E-02
negative regulation of multi-organism process (GO:0043901)	11.99	4.03E-06
response to virus (GO:0009615)	10.4	1.99E-07
regulation of viral life cycle (GO:1903900)	9.86	2.91E-03
regulation of viral process (GO:0050792)	9.26	4.86E-03
regulation of symbiosis, encompassing mutualism through parasitism (GO:0043903)	8.56	2.39E-03
positive regulation of ERK1 and ERK2 cascade (GO:0070374)	7.77	2.01E-02

immune effector process (GO:0002252)	7.16	2.79E-06
regulation of ERK1 and ERK2 cascade (GO:0070372)	6.92	4.97E-03
innate immune response (GO:0045087)	5.7	5.79E-04
defense response to other organism (GO:0098542)	5.38	4.67E-04
response to biotic stimulus (GO:0009607)	5.08	1.06E-07
regulation of multi-organism process (GO:0043900)	5.02	6.95E-03
defense response (GO:0006952)	4.72	1.57E-08
response to other organism (GO:0051707)	4.56	3.07E-05
response to external biotic stimulus (GO:0043207)	4.55	3.19E-05
response to cytokine (GO:0034097)	4.23	2.43E-03
immune response (GO:0006955)	4.21	1.25E-05
cell migration (GO:0016477)	3.74	1.33E-02
immune system process (GO:0002376)	3.6	1.23E-08
localization of cell (GO:0051674)	3.52	1.67E-02
cell motility (GO:0048870)	3.52	1.67E-02
response to external stimulus (GO:0009605)	3.46	5.38E-07
movement of cell or subcellular component (GO:0006928)	2.87	4.36E-02
multi-organism process (GO:0051704)	2.61	3.69E-02
response to stress (GO:0006950)	2.43	2.87E-04
Unclassified (UNCLASSIFIED)	0.79	0.00E+00

(a)

**Items**	**fold Enrichment**	**P-value**
TAP2 binding (GO:0046979)	> 100	9.77E-03

TAP binding (GO:0046977)	> 100	9.77E-03
fatty acid alpha-hydroxylase activity (GO:0080132)	> 100	9.77E-03
UMP kinase activity (GO:0033862)	> 100	9.77E-03
lysine-tRNA ligase activity (GO:0004824)	> 100	9.77E-03
double-stranded RNA adenosine deaminase activity (GO:0003726)	> 100	1.89E-04
thymidylate kinase activity (GO:0004798)	> 100	9.77E-03
steroid delta-isomerase activity (GO:0004769)	> 100	9.77E-03
spermidine synthase activity (GO:0004766)	> 100	9.77E-03
3-hydroxyisobutyrate dehydrogenase activity (GO:0008442)	> 100	9.77E-03
morphogen activity (GO:0016015)	> 100	9.77E-03
interleukin-8 receptor binding (GO:0005153)	> 100	9.77E-03
delta24(24-1) sterol reductase activity (GO:0000246)	> 100	9.77E-03
Mo-molybdopterin cofactor sulfurase activity (GO:0008265)	> 100	9.77E-03
ISG15-specific protease activity (GO:0019785)	> 100	9.77E-03
ISG15 activating enzyme activity (GO:0019782)	> 100	9.77E-03
CCR2 chemokine receptor binding (GO:0031727)	> 100	9.77E-03
1-pyrroline-5-carboxylate dehydrogenase activity (GO:0003842)	50.95	1.94E-02
citrate synthase activity (GO:0036440)	50.95	1.94E-02
citrate (Si)-synthase activity (GO:0004108)	50.95	1.94E-02
extracellular matrix protein binding (GO:1990430)	50.95	1.94E-02
ISG15 transferase activity (GO:0042296)	50.95	7.47E-04
phosphogluconate dehydrogenase (decarboxylating) activity (GO:0004616)	50.95	7.47E-04
hydroxymethylglutaryl-CoA synthase activity (GO:0004421)	50.95	1.94E-02
cobalt ion binding (GO:0050897)	50.95	1.94E-02

2'-5'-oligoadenylate synthetase activity (GO:0001730)	50.95	1.94E-02
RAGE receptor binding (GO:0050786)	40.76	1.16E-03
uridylate kinase activity (GO:0009041)	33.97	2.90E-02
ubiquitin activating enzyme activity (GO:0004839)	33.97	2.90E-02
single-stranded DNA 3'-5' exodeoxyribonuclease activity (GO:0008310)	33.97	2.90E-02
peptide-transporting ATPase activity (GO:0015440)	33.97	2.90E-02
Toll-like receptor 4 binding (GO:0035662)	33.97	2.90E-02
transferase activity, transferring acyl groups, acyl groups converted into alkyl on transfer (GO:0046912)	29.11	2.24E-03
peptide antigen binding (GO:0042605)	27.17	1.69E-05
6-phosphofructo-2-kinase activity (GO:0003873)	25.47	3.85E-02
phosphatidylglycerol binding (GO:1901611)	25.47	3.85E-02
arachidonic acid binding (GO:0050544)	25.47	3.85E-02
icosatetraenoic acid binding (GO:0050543)	25.47	3.85E-02
icosanoid binding (GO:0050542)	25.47	3.85E-02
fibroblast growth factor-activated receptor activity (GO:0005007)	25.47	3.85E-02
beta-N-acetylhexosaminidase activity (GO:0004563)	25.47	3.85E-02
3-beta-hydroxy-delta5-steroid dehydrogenase activity (GO:0003854)	20.38	4.79E-02
peptide transporter activity (GO:0015197)	20.38	4.79E-02
cytidylate kinase activity (GO:0004127)	20.38	4.79E-02
natural killer cell lectin-like receptor binding (GO:0046703)	20.38	4.79E-02
sphingolipid transporter activity (GO:0046624)	20.38	4.79E-02
long-chain fatty acid binding (GO:0036041)	20.38	4.79E-02
opsonin binding (GO:0001846)	20.38	4.79E-02
adenosine deaminase activity (GO:0004000)	18.53	5.40E-03

MAP kinase tyrosine/serine/threonine phosphatase activity (GO:0017017)	15.68	7.45E-03
MAP kinase phosphatase activity (GO:0033549)	14.56	8.58E-03
calcium-transporting ATPase activity (GO:0005388)	13.59	9.79E-03
CCR chemokine receptor binding (GO:0048020)	13.29	1.60E-03
antigen binding (GO:0003823)	12.13	6.77E-05
extracellular matrix binding (GO:0050840)	11.99	3.87E-04
chemokine activity (GO:0008009)	11.02	5.30E-04
insulin-like growth factor binding (GO:0005520)	10.92	2.79E-03
R-SMAD binding (GO:0070412)	10.73	1.53E-02
chemokine receptor binding (GO:0042379)	9.94	7.75E-04
protein kinase C binding (GO:0005080)	9.26	2.01E-02
double-stranded RNA binding (GO:0003725)	9.06	1.09E-03
ubiquitin conjugating enzyme activity (GO:0061631)	8.86	2.19E-02
chemoattractant activity (GO:0042056)	8.86	2.19E-02
ubiquitin-like protein conjugating enzyme activity (GO:0061650)	8.49	2.37E-02
deaminase activity (GO:0019239)	8.49	2.37E-02
single-stranded RNA binding (GO:0003727)	8.04	6.48E-03
protein tyrosine/serine/threonine phosphatase activity (GO:0008138)	7.64	7.46E-03
hydrolase activity, acting on carbon-nitrogen (but not peptide) bonds, in cyclic amidines (GO:0016814)	7.55	2.94E-02
PDZ domain binding (GO:0030165)	7.11	9.07E-03
integrin binding (GO:0005178)	6.27	4.08E-03
SMAD binding (GO:0046332)	5.99	1.43E-02
protease binding (GO:0002020)	5.91	5.03E-03
ATPase activity, coupled to transmembrane movement of ions, phosphorylative mechanism (GO:0015662)	5.82	4.69E-02

cation-transporting ATPase activity (GO:0019829)	5.18	2.09E-02
ATPase coupled ion transmembrane transporter activity (GO:0042625)	5.18	2.09E-02
growth factor binding (GO:0019838)	4.85	9.87E-03
hydrolase activity, acting on acid anhydrides, catalyzing transmembrane movement of substances (GO:0016820)	4.7	1.95E-03
heparin binding (GO:0008201)	4.38	1.39E-02
cell adhesion molecule binding (GO:0050839)	4.25	6.99E-03
ubiquitin-like protein transferase activity (GO:0019787)	4.21	8.26E-06
ATPase activity, coupled to transmembrane movement of substances (GO:0042626)	4.08	8.24E-03
ubiquitin-protein transferase activity (GO:0004842)	4.05	2.58E-05
P-P-bond-hydrolysis-driven transmembrane transporter activity (GO:0015405)	3.92	9.64E-03
primary active transmembrane transporter activity (GO:0015399)	3.92	9.64E-03
cytokine activity (GO:0005125)	3.88	2.47E-03
oxidoreductase activity, acting on CH-OH group of donors (GO:0016614)	3.51	2.83E-02
ATPase activity, coupled to movement of substances (GO:0043492)	3.42	1.65E-02
glycosaminoglycan binding (GO:0005539)	3.31	3.39E-02
ligase activity (GO:0016874)	2.6	4.52E-02
receptor binding (GO:0005102)	2.16	6.10E-04
zinc ion binding (GO:0008270)	1.9	7.40E-03
RNA binding (GO:0003723)	1.89	2.60E-03
poly(A) RNA binding (GO:0044822)	1.85	1.42E-02
transition metal ion binding (GO:0046914)	1.69	1.55E-02
enzyme binding (GO:0019899)	1.64	2.38E-02
protein binding (GO:0005515)	1.43	1.54E-03
nucleic acid binding (GO:0003676)	1.31	4.34E-02

binding (GO:0005488)	1.28	1.02E-04
heterocyclic compound binding (GO:1901363)	1.24	3.94E-02
organic cyclic compound binding (GO:0097159)	1.22	4.86E-02
molecular_function (GO:0003674)	1.11	1.09E-02
Unclassified (UNCLASSIFIED)	0.76	1.09E-02
signal transducer activity (GO:0004871)	< 0.2	6.87E-06
receptor activity (GO:0004872)	< 0.2	2.06E-06
molecular transducer activity (GO:0060089)	< 0.2	2.06E-06
transmembrane signaling receptor activity (GO:0004888)	< 0.2	3.14E-06
transmembrane receptor activity (GO:0099600)	< 0.2	2.15E-06
signaling receptor activity (GO:0038023)	< 0.2	1.42E-06

(b)

**GO cellular component complete**	**fold Enrichment**	**P-value**
glycogen granule (GO:0042587)	> 100	9.77E-03
integrin alphav-beta8 complex (GO:0034686)	> 100	9.77E-03
integrin alphav-beta3 complex (GO:0034683)	> 100	9.77E-03
integrin alpha8-beta1 complex (GO:0034678)	> 100	9.77E-03
supraspliceosomal complex (GO:0044530)	50.95	1.94E-02
alphav-beta3 integrin-IGF-1-IGF1R complex (GO:0035867)	50.95	1.94E-02
integrin alphav-beta5 complex (GO:0034684)	50.95	1.94E-02
TAP complex (GO:0042825)	50.95	1.94E-02
cytoplasmic side of apical plasma membrane (GO:0098592)	50.95	1.94E-02
trans-Golgi network transport vesicle membrane (GO:0012510)	40.76	1.16E-03

clathrin coat of trans-Golgi network vesicle (GO:0030130)	40.76	1.16E-03
extrinsic component of endoplasmic reticulum membrane (GO:0042406)	33.97	2.90E-02
extrinsic component of external side of plasma membrane (GO:0031232)	33.97	2.90E-02
actin cap (GO:0030478)	33.97	2.90E-02
TCR signalosome (GO:0036398)	33.97	2.90E-02
terminal web (GO:1990357)	33.97	2.90E-02
MHC class I peptide loading complex (GO:0042824)	33.97	2.90E-02
MHC class I protein complex (GO:0042612)	30.57	1.44E-04
smooth muscle contractile fiber (GO:0030485)	25.47	3.85E-02
cytolytic granule (GO:0044194)	25.47	3.85E-02
NLRP3 inflammasome complex (GO:0072559)	25.47	3.85E-02
clathrin coat of coated pit (GO:0030132)	22.64	3.66E-03
inflammasome complex (GO:0061702)	20.38	4.79E-02
MHC protein complex (GO:0042611)	20.38	5.15E-05
ciliary transition fiber (GO:0097539)	20.38	4.79E-02
intercellular canaliculus (GO:0046581)	20.38	4.79E-02
trans-Golgi network transport vesicle (GO:0030140)	18.53	5.40E-03
clathrin vesicle coat (GO:0030125)	18.53	5.40E-03
clathrin-coated vesicle membrane (GO:0030665)	14.56	8.58E-03
high-density lipoprotein particle (GO:0034364)	13.59	9.79E-03
clathrin coat (GO:0030118)	12.74	3.08E-04
integrin complex (GO:0008305)	12.74	1.81E-03
protein complex involved in cell adhesion (GO:0098636)	12.74	1.81E-03
ruffle membrane (GO:0032587)	12.35	3.46E-04

vacuolar proton-transporting V-type ATPase complex (GO:0016471)	11.99	1.24E-02
lipoprotein particle (GO:1990777)	9.7	1.85E-02
plasma lipoprotein particle (GO:0034358)	9.7	1.85E-02
clathrin adaptor complex (GO:0030131)	8.86	2.19E-02
protein-lipid complex (GO:0032994)	8.86	2.19E-02
proton-transporting V-type ATPase complex (GO:0033176)	8.49	2.37E-02
Golgi-associated vesicle membrane (GO:0030660)	8.49	2.37E-02
ruffle (GO:0001726)	7.84	1.38E-04
leading edge membrane (GO:0031256)	7.41	2.26E-03
clathrin-coated pit (GO:0005905)	7.28	3.14E-02
cortical actin cytoskeleton (GO:0030864)	7.28	3.14E-02
filopodium (GO:0030175)	6.5	1.15E-02
vesicle coat (GO:0030120)	6.37	4.00E-02
AP-type membrane coat adaptor complex (GO:0030119)	6.18	4.23E-02
microvillus (GO:0005902)	6.18	4.23E-02
clathrin-coated vesicle (GO:0030136)	6.11	1.36E-02
postsynaptic density (GO:0097481)	5.99	4.46E-02
membrane coat (GO:0030117)	5.58	6.12E-03
coated membrane (GO:0048475)	5.58	6.12E-03
cell projection membrane (GO:0031253)	4.81	4.19E-03
cell leading edge (GO:0031252)	4.36	5.87E-04
proteinaceous extracellular matrix (GO:0005578)	3.99	5.10E-04
extracellular matrix (GO:0031012)	3.89	7.65E-05
external side of plasma membrane (GO:0009897)	3.35	1.78E-02

apical plasma membrane (GO:0016324)	3.06	4.31E-02
apical part of cell (GO:0045177)	2.89	3.08E-02
side of membrane (GO:0098552)	2.65	1.78E-02
plasma membrane region (GO:0098590)	2.49	7.68E-03
plasma membrane protein complex (GO:0098797)	2.46	1.21E-02
actin cytoskeleton (GO:0015629)	2.35	3.11E-02
lysosome (GO:0005764)	2.35	4.45E-02
lytic vacuole (GO:0000323)	2.33	4.59E-02
endoplasmic reticulum membrane (GO:0005789)	2.27	2.65E-02
nuclear outer membrane-endoplasmic reticulum membrane network (GO:0042175)	2.23	2.92E-02
cell surface (GO:0009986)	2.02	3.65E-02
extracellular space (GO:0005615)	2.01	4.17E-03
membrane protein complex (GO:0098796)	1.84	1.77E-02
bounding membrane of organelle (GO:0098588)	1.77	1.67E-02
endoplasmic reticulum (GO:0005783)	1.74	3.27E-02
extracellular region part (GO:0044421)	1.71	2.58E-04
extracellular region (GO:0005576)	1.7	1.15E-04
organelle membrane (GO:0031090)	1.6	1.98E-02
vesicle (GO:0031982)	1.55	6.15E-03
extracellular vesicle (GO:1903561)	1.54	1.55E-02
extracellular organelle (GO:0043230)	1.53	1.56E-02
plasma membrane part (GO:0044459)	1.53	2.57E-02
extracellular exosome (GO:0070062)	1.49	2.42E-02
membrane-bounded vesicle (GO:0031988)	1.46	1.89E-02

cytoplasm (GO:0005737)	1.33	8.51E-04
membrane-bounded organelle (GO:0043227)	1.31	3.27E-04
intracellular membrane-bounded organelle (GO:0043231)	1.29	1.69E-03
nucleus (GO:0005634)	1.28	2.18E-02
organelle (GO:0043226)	1.25	1.14E-03
cytoplasmic part (GO:0044444)	1.24	4.09E-02
intracellular organelle (GO:0043229)	1.24	4.49E-03
intracellular part (GO:0044424)	1.18	1.06E-02
intracellular (GO:0005622)	1.17	8.69E-03
cellular_component (GO:0005575)	1.11	5.92E-03
Unclassified (UNCLASSIFIED)	0.71	5.92E-03
neuron part (GO:0097458)	< 0.2	1.74E-02

(c)

Supplementary Table S6. Pathway analysis for DEGs.

Pathway ID	Pathway	DEGs with pathway annotation	All genes with pathway annotation	Pvalue	Qvalue
ko05162	Measles	17 (8.37%)	343 (1.78%)	1.41E-07	1.76E-05
ko05160	Hepatitis C	14 (6.9%)	237 (1.23%)	2.26E-07	1.76E-05
ko04622	RIG-I-like receptor signaling pathway	9 (4.43%)	132 (0.68%)	1.09E-05	5.68E-04
ko04142	Lysosome	10 (4.93%)	210 (1.09%)	7.91E-05	3.08E-03
ko05323	Rheumatoid arthritis	9 (4.43%)	176 (0.91%)	0.000105086	3.28E-03
ko04120	Ubiquitin mediated proteolysis	10 (4.93%)	251 (1.3%)	0.000336585	8.75E-03
ko04621	NOD-like receptor signaling pathway	7 (3.45%)	135 (0.7%)	0.000569626	1.27E-02

ko04612	Antigen processing and presentation	8 (3.94%)	200 (1.04%)	0.001283502	2.50E-02
ko04623	Cytosolic DNA-sensing pathway	6 (2.96%)	131 (0.68%)	0.002640554	4.58E-02
ko04062	Chemokine signaling pathway	11 (5.42%)	393 (2.04%)	0.00305503	4.77E-02
ko04620	Toll-like receptor signaling pathway	7 (3.45%)	186 (0.96%)	0.00358754	5.09E-02
ko05200	Pathways in cancer	16 (7.88%)	720 (3.73%)	0.004001136	5.20E-02
ko05120	Epithelial cell signaling in Helicobacter pylori infection	5 (2.46%)	108 (0.56%)	0.005684931	6.82E-02
ko04060	Cytokine-cytokine receptor interaction	10 (4.93%)	381 (1.97%)	0.007228815	8.05E-02
ko05215	Prostate cancer	6 (2.96%)	170 (0.88%)	0.009272729	9.64E-02
ko05222	Small cell lung cancer	6 (2.96%)	213 (1.1%)	0.02534562	2.24E-01
ko04115	p53 signaling pathway	5 (2.46%)	161 (0.83%)	0.02777991	2.24E-01
ko05142	Chagas disease (American trypanosomiasis)	6 (2.96%)	219 (1.13%)	0.02851735	2.24E-01
ko04510	Focal adhesion	19 (9.36%)	1138 (5.89%)	0.03127463	2.24E-01
ko04978	Mineral absorption	4 (1.97%)	113 (0.59%)	0.03146964	2.24E-01
ko04380	Osteoclast differentiation	6 (2.96%)	227 (1.18%)	0.03313876	2.24E-01
ko05330	Allograft rejection	4 (1.97%)	116 (0.6%)	0.03416949	2.24E-01
ko04662	B cell receptor signaling pathway	5 (2.46%)	173 (0.9%)	0.03620516	2.24E-01
ko05332	Graft-versus-host disease	4 (1.97%)	119 (0.62%)	0.03700024	2.24E-01
ko00100	Steroid biosynthesis	2 (0.99%)	29 (0.15%)	0.037074	2.24E-01
ko05218	Melanoma	4 (1.97%)	120 (0.62%)	0.03797298	2.24E-01
ko04940	Type I diabetes mellitus	4 (1.97%)	121 (0.63%)	0.03896032	2.24E-01
ko04610	Complement and coagulation cascades	5 (2.46%)	178 (0.92%)	0.04013292	2.24E-01
ko00480	Glutathione metabolism	3 (1.48%)	76 (0.39%)	0.04602105	2.47E-01
ko04650	Natural killer cell mediated cytotoxicity	6 (2.96%)	248 (1.28%)	0.04749406	2.47E-01
ko05012	Parkinson's disease	5 (2.46%)	196 (1.02%)	0.05635147	2.70E-01
ko00280	Valine, leucine and isoleucine degradation	3 (1.48%)	83 (0.43%)	0.05707608	2.70E-01

ko05214	Glioma	4 (1.97%)	138 (0.71%)	0.05797451	2.70E-01
ko05219	Bladder cancer	3 (1.48%)	84 (0.44%)	0.05874931	2.70E-01
ko00030	Pentose phosphate pathway	2 (0.99%)	42 (0.22%)	0.07200163	3.14E-01
ko03320	PPAR signaling pathway	4 (1.97%)	149 (0.77%)	0.0724898	3.14E-01
ko04110	Cell cycle	5 (2.46%)	218 (1.13%)	0.0806111	3.32E-01
ko05130	Pathogenic Escherichia coli infection	6 (2.96%)	285 (1.48%)	0.08096093	3.32E-01
ko04144	Endocytosis	9 (4.43%)	501 (2.59%)	0.08309833	3.32E-01
ko04145	Phagosome	7 (3.45%)	359 (1.86%)	0.08547158	3.33E-01
ko00630	Glyoxylate and dicarboxylate metabolism	2 (0.99%)	47 (0.24%)	0.0874161	3.33E-01
ko05213	Endometrial cancer	3 (1.48%)	102 (0.53%)	0.09261076	3.44E-01
ko05320	Autoimmune thyroid disease	4 (1.97%)	166 (0.86%)	0.09817875	3.56E-01
ko04512	ECM-receptor interaction	13 (6.4%)	841 (4.36%)	0.1069662	3.79E-01
ko05131	Shigellosis	5 (2.46%)	247 (1.28%)	0.1197637	4.15E-01
ko04966	Collecting duct acid secretion	2 (0.99%)	58 (0.3%)	0.124227	4.17E-01
ko05140	Leishmaniasis	3 (1.48%)	117 (0.61%)	0.125649	4.17E-01
ko05145	Toxoplasmosis	5 (2.46%)	253 (1.31%)	0.128815	4.19E-01
ko05143	African trypanosomiasis	2 (0.99%)	62 (0.32%)	0.1384028	4.41E-01
ko05216	Thyroid cancer	2 (0.99%)	63 (0.33%)	0.1420005	4.41E-01
ko04141	Protein processing in endoplasmic reticulum	6 (2.96%)	341 (1.77%)	0.1510239	4.41E-01
ko00380	Tryptophan metabolism	2 (0.99%)	66 (0.34%)	0.1529096	4.41E-01
ko00020	Citrate cycle (TCA cycle)	2 (0.99%)	66 (0.34%)	0.1529096	4.41E-01
ko00790	Folate biosynthesis	1 (0.49%)	16 (0.08%)	0.1556257	4.41E-01
ko00072	Synthesis and degradation of ketone bodies	1 (0.49%)	16 (0.08%)	0.1556257	4.41E-01
ko00603	Glycosphingolipid biosynthesis - globo series	1 (0.49%)	17 (0.09%)	0.1645097	4.58E-01
ko05020	Prion diseases	3 (1.48%)	134 (0.69%)	0.1673345	4.58E-01
ko04010	MAPK signaling pathway	8 (3.94%)	514 (2.66%)	0.1751208	4.71E-01

ko04810	Regulation of actin cytoskeleton	11 (5.42%)	771 (3.99%)	0.1896771	4.96E-01
ko04711	Circadian rhythm - fly	1 (0.49%)	20 (0.1%)	0.1906075	4.96E-01
ko04670	Leukocyte transendothelial migration	5 (2.46%)	294 (1.52%)	0.1981405	5.07E-01
ko00604	Glycosphingolipid biosynthesis - ganglio series	1 (0.49%)	24 (0.12%)	0.2241469	5.55E-01
ko00450	Selenocompound metabolism	1 (0.49%)	24 (0.12%)	0.2241469	5.55E-01
ko00330	Arginine and proline metabolism	2 (0.99%)	88 (0.46%)	0.2364979	5.57E-01
ko04640	Hematopoietic cell lineage	3 (1.48%)	160 (0.83%)	0.2372311	5.57E-01
ko05110	Vibrio cholerae infection	5 (2.46%)	315 (1.63%)	0.2377764	5.57E-01
ko00670	One carbon pool by folate	1 (0.49%)	27 (0.14%)	0.2483905	5.57E-01
ko04614	Renin-angiotensin system	1 (0.49%)	27 (0.14%)	0.2483905	5.57E-01
ko00511	Other glycan degradation	1 (0.49%)	28 (0.15%)	0.256303	5.57E-01
ko04012	ErbB signaling pathway	3 (1.48%)	167 (0.86%)	0.2568798	5.57E-01
ko04350	TGF-beta signaling pathway	3 (1.48%)	168 (0.87%)	0.2597058	5.57E-01
ko05340	Primary immunodeficiency	2 (0.99%)	94 (0.49%)	0.2598502	5.57E-01
ko00531	Glycosaminoglycan degradation	1 (0.49%)	29 (0.15%)	0.2641326	5.57E-01
ko00900	Terpenoid backbone biosynthesis	1 (0.49%)	29 (0.15%)	0.2641326	5.57E-01
ko05416	Viral myocarditis	6 (2.96%)	416 (2.15%)	0.2738435	5.70E-01
ko02010	ABC transporters	2 (0.99%)	99 (0.51%)	0.2793345	5.73E-01
ko05310	Asthma	2 (0.99%)	102 (0.53%)	0.2910113	5.75E-01
ko00190	Oxidative phosphorylation	3 (1.48%)	179 (0.93%)	0.2910244	5.75E-01
ko03060	Protein export	1 (0.49%)	33 (0.17%)	0.2946393	5.75E-01
ko04950	Maturity onset diabetes of the young	1 (0.49%)	33 (0.17%)	0.2946393	5.75E-01
ko01040	Biosynthesis of unsaturated fatty acids	1 (0.49%)	35 (0.18%)	0.3094173	5.96E-01
ko00360	Phenylalanine metabolism	1 (0.49%)	36 (0.19%)	0.3166904	6.02E-01
ko05221	Acute myeloid leukemia	2 (0.99%)	110 (0.57%)	0.3220092	6.05E-01
ko05100	Bacterial invasion of epithelial cells	4 (1.97%)	282 (1.46%)	0.3447081	6.19E-01

ko00340	Histidine metabolism	1 (0.49%)	40 (0.21%)	0.3450283	6.19E-01
ko00250	Alanine, aspartate and glutamate metabolism	1 (0.49%)	40 (0.21%)	0.3450283	6.19E-01
ko05210	Colorectal cancer	2 (0.99%)	116 (0.6%)	0.3450423	6.19E-01
ko05217	Basal cell carcinoma	2 (0.99%)	119 (0.62%)	0.3564664	6.32E-01
ko04514	Cell adhesion molecules (CAMs)	4 (1.97%)	292 (1.51%)	0.3685591	6.36E-01
ko04520	Adherens junction	4 (1.97%)	292 (1.51%)	0.3685591	6.36E-01
ko04722	Neurotrophin signaling pathway	4 (1.97%)	293 (1.52%)	0.3709431	6.36E-01
ko05146	Amoebiasis	12 (5.91%)	1012 (5.24%)	0.3762386	6.38E-01
ko04310	Wnt signaling pathway	4 (1.97%)	300 (1.55%)	0.3876114	6.50E-01
ko00410	beta-Alanine metabolism	1 (0.49%)	47 (0.24%)	0.3918341	6.50E-01
ko05212	Pancreatic cancer	2 (0.99%)	131 (0.68%)	0.4013849	6.57E-01
ko00650	Butanoate metabolism	1 (0.49%)	49 (0.25%)	0.404585	6.57E-01
ko05322	Systemic lupus erythematosus	3 (1.48%)	231 (1.2%)	0.4389462	7.06E-01
ko00350	Tyrosine metabolism	1 (0.49%)	57 (0.3%)	0.4529828	7.21E-01
ko05220	Chronic myeloid leukemia	2 (0.99%)	149 (0.77%)	0.4658311	7.26E-01
ko00270	Cysteine and methionine metabolism	1 (0.49%)	60 (0.31%)	0.4701048	7.26E-01
ko00051	Fructose and mannose metabolism	1 (0.49%)	60 (0.31%)	0.4701048	7.26E-01
ko05144	Malaria	2 (0.99%)	155 (0.8%)	0.4863988	7.44E-01
ko04540	Gap junction	2 (0.99%)	160 (0.83%)	0.5031599	7.57E-01
ko01100	Metabolic pathways	19 (9.36%)	1778 (9.21%)	0.5056	7.57E-01
ko00860	Porphyrin and chlorophyll metabolism	1 (0.49%)	69 (0.36%)	0.5183363	7.57E-01
ko00140	Steroid hormone biosynthesis	1 (0.49%)	69 (0.36%)	0.5183363	7.57E-01
ko04630	Jak-STAT signaling pathway	3 (1.48%)	261 (1.35%)	0.5192132	7.57E-01
ko00520	Amino sugar and nucleotide sugar metabolism	1 (0.49%)	72 (0.37%)	0.533422	7.70E-01
ko04977	Vitamin digestion and absorption	1 (0.49%)	74 (0.38%)	0.543217	7.76E-01
ko04260	Cardiac muscle contraction	3 (1.48%)	272 (1.41%)	0.5470793	7.76E-01

ko05150	Staphylococcus aureus infection	2 (0.99%)	178 (0.92%)	0.5605084	7.88E-01
ko04960	Aldosterone-regulated sodium reabsorption	1 (0.49%)	79 (0.41%)	0.5668185	7.88E-01
ko04962	Vasopressin-regulated water reabsorption	1 (0.49%)	80 (0.41%)	0.5713911	7.88E-01
ko00510	N-Glycan biosynthesis	1 (0.49%)	81 (0.42%)	0.5759156	7.88E-01
ko04974	Protein digestion and absorption	8 (3.94%)	787 (4.08%)	0.588984	7.94E-01
ko05412	Arrhythmogenic right ventricular cardiomyopathy (ARVC)	2 (0.99%)	188 (0.97%)	0.5902786	7.94E-01
ko04972	Pancreatic secretion	2 (0.99%)	196 (1.02%)	0.6130025	8.13E-01
ko00970	Aminoacyl-tRNA biosynthesis	1 (0.49%)	91 (0.47%)	0.6186275	8.13E-01
ko04340	Hedgehog signaling pathway	1 (0.49%)	93 (0.48%)	0.6266417	8.13E-01
ko05223	Non-small cell lung cancer	1 (0.49%)	95 (0.49%)	0.6344882	8.13E-01
ko04973	Carbohydrate digestion and absorption	1 (0.49%)	95 (0.49%)	0.6344882	8.13E-01
ko04970	Salivary secretion	4 (1.97%)	414 (2.14%)	0.6360182	8.13E-01
ko04916	Melanogenesis	2 (0.99%)	207 (1.07%)	0.642665	8.15E-01
ko04530	Tight junction	4 (1.97%)	432 (2.24%)	0.6689051	8.42E-01
ko04330	Notch signaling pathway	1 (0.49%)	111 (0.57%)	0.6916307	8.63E-01
ko00562	Inositol phosphate metabolism	1 (0.49%)	116 (0.6%)	0.7075939	8.73E-01
ko04672	Intestinal immune network for IgA production	1 (0.49%)	117 (0.61%)	0.7106864	8.73E-01
ko03040	Spliceosome	3 (1.48%)	352 (1.82%)	0.7188033	8.76E-01
ko05211	Renal cell carcinoma	1 (0.49%)	122 (0.63%)	0.7256677	8.78E-01
ko05410	Hypertrophic cardiomyopathy (HCM)	3 (1.48%)	369 (1.91%)	0.7480053	8.93E-01
ko05414	Dilated cardiomyopathy	3 (1.48%)	370 (1.92%)	0.7496455	8.93E-01
ko00564	Glycerophospholipid metabolism	1 (0.49%)	135 (0.7%)	0.7611036	8.95E-01
ko04660	T cell receptor signaling pathway	2 (0.99%)	262 (1.36%)	0.7647435	8.95E-01
ko04320	Dorso-ventral axis formation	1 (0.49%)	138 (0.71%)	0.7686113	8.95E-01
ko04370	VEGF signaling pathway	1 (0.49%)	144 (0.75%)	0.7829296	9.05E-01

ko03015	mRNA surveillance pathway	3 (1.48%)	397 (2.06%)	0.7907879	9.06E-01
ko04664	Fc epsilon RI signaling pathway	1 (0.49%)	151 (0.78%)	0.7985235	9.06E-01
ko04976	Bile secretion	1 (0.49%)	154 (0.8%)	0.8048605	9.06E-01
ko04210	Apoptosis	1 (0.49%)	157 (0.81%)	0.810999	9.06E-01
ko04910	Insulin signaling pathway	2 (0.99%)	291 (1.51%)	0.8133092	9.06E-01
ko04666	Fc gamma R-mediated phagocytosis	2 (0.99%)	307 (1.59%)	0.8361095	9.13E-01
ko05016	Huntington's disease	3 (1.48%)	438 (2.27%)	0.8424462	9.13E-01
ko05010	Alzheimer's disease	2 (0.99%)	313 (1.62%)	0.8439933	9.13E-01
ko00310	Lysine degradation	1 (0.49%)	177 (0.92%)	0.8472906	9.13E-01
ko00240	Pyrimidine metabolism	1 (0.49%)	178 (0.92%)	0.8489108	9.13E-01
ko05014	Amyotrophic lateral sclerosis (ALS)	1 (0.49%)	187 (0.97%)	0.8627443	9.22E-01
ko04070	Phosphatidylinositol signaling system	1 (0.49%)	198 (1.03%)	0.8779513	9.31E-01
ko04360	Axon guidance	2 (0.99%)	348 (1.8%)	0.8835122	9.31E-01
ko04912	GnRH signaling pathway	1 (0.49%)	213 (1.1%)	0.8960214	9.38E-01
ko04114	Oocyte meiosis	1 (0.49%)	221 (1.14%)	0.9045432	9.41E-01
ko04971	Gastric acid secretion	1 (0.49%)	238 (1.23%)	0.9204135	9.51E-01
ko03013	RNA transport	3 (1.48%)	557 (2.88%)	0.9349765	9.56E-01
ko04020	Calcium signaling pathway	2 (0.99%)	420 (2.18%)	0.937452	9.56E-01
ko03010	Ribosome	1 (0.49%)	281 (1.46%)	0.949791	9.62E-01
ko04270	Vascular smooth muscle contraction	2 (0.99%)	465 (2.41%)	0.9580951	9.64E-01
ko04080	Neuroactive ligand-receptor interaction	1 (0.49%)	359 (1.86%)	0.9782868	9.78E-01
